# Associations of physical activity domains and delayed biological aging: assessing heterogeneity and interaction of effects

**DOI:** 10.5114/biolsport.2026.158670

**Published:** 2026-02-06

**Authors:** Yongyu Huang, Yang Wang, Yanwei You, Ming Ding, Zuosheng Lu, Qing Wang

**Affiliations:** 1 School of Physical Education and Sports Science, South China Normal University, Guangzhou, China; 2 Division of Sports Science & Physical Education, Tsinghua University, Beijing 100084, China; 3 Department of Epidemiology, Center for Global Health, School of Public Health, Nanjing Medical University, Nanjing, 211166 China; 4 Epidemiology and Statistics, Institute of Basic Medical Sciences, Chinese Academy of Medical Sciences & School of Basic Medicine, Peking Union Medical College, Beijing, China; 5 State Key Laboratory of Common Mechanism Research for Major Diseases, Beijing, China

**Keywords:** Physical activity, Biological Aging, Interaction of Effects, Associations, Public Health

## Abstract

Physical activity (PA) is widely recognized for its physical and mental health benefits, but the effects of different PA domains on delayed biological aging remain inconclusive. This study aimed to investigate the specific effects of occupational PA (OPA), transportation PA (TPA), and leisure-time PA (LTPA) on delayed biological aging, as well as the heterogeneity and joint interactions of these effects. We analyzed data from 18,362 adults in NHANES 2007–2010 and 2015–2018. Biological aging was assessed using Klemera–Doubal Method Biological Age (KDM-BA), Phenotypic Age (PhenoAge), and Homeostatic Dysregulation (HD). Delayed aging was defined as biological age acceleration < 0 for KDM-BA and PhenoAge, and as values below the median for HD. Logistic regression models were applied to estimate associations of PA domains and levels with delayed aging, and joint analyses were conducted to examine combined effects. Results showed that high OPA was associated with a lower likelihood of delayed aging (KDM, OR=0.84, 95% CI=0.78–0.91; PhenoAge, OR=0.86, 95% CI=0.79–0.94), whereas high LTPA (PhenoAge, OR=1.35, 95% CI=1.22–1.49; HD, OR=1.18, 95% CI=1.09–1.29) was associated with a higher likelihood, while associations for TPA were not significant after adjustment. Joint analysis showed that combinations with high OPA were associated with lower likelihood of delayed aging, whereas those with high LTPA were associated with higher likelihood. These findings highlight the distinct roles of different PA domains and levels in delaying biological aging, providing important evidence for public health strategies to promote healthy aging.

## INTRODUCTION

Aging is characterized by the progressive loss of physiological integrity, resulting in functional decline and increased mortality risk. This decline is a major risk factor for many human diseases [1]. To quantify this process, gerontology has developed several approaches to measuring biological aging. These methods range from individual biomarkers, such as telomere length, to algorithms that integrate epigenetic, proteomic, metabolomic, and other molecular data [2, 3]. Among these, algorithms that combine standard clinical parameters have demonstrated the highest accuracy in predicting morbidity and mortality [4, 5]. Biological age provides a more accurate representation of an individual’s aging status than chronological age, which is only a temporal marker and does not reflect health status. Individuals with the same chronological age may exhibit markedly different biological ages [6]. These measures of biological aging are therefore of great importance in public health and geriatric medicine, as they provide actionable indicators for identifying individuals at higher risk and for developing strategies to promote healthy aging.

Evidence from animal and human studies suggests that biological aging is modifiable [7, 8]. Slowing biological aging may prevent or delay the onset of a range of age-related diseases and potentially extend lifespan [7]. Research indicates that a composite healthy lifestyle index—encompassing non-smoking, non-drinking, sufficient physical activity, and a balanced diet—is strongly associated with biochemical markers of aging [9, 10]. The duration of physical activity is closely linked to health. According to the World Health Organization (WHO) 2020 guidelines on physical activity and sedentary behaviour [11], adequate physical activity for adults is defined as 150–300 minutes of moderate-intensity aerobic activity per week, 75–150 minutes of vigorous-intensity aerobic activity per week, or an equivalent combination of both. In addition, adults are advised to engage in muscle-strengthening activities involving major muscle groups on two or more days per week. The guidelines further emphasize that “some physical activity is better than none” and that adults should “move more and sit less” throughout the day, as reducing sedentary behaviour also confers substantial health benefits. Epidemiological studies indicate that adequate physical activity reduces all-cause mortality and the incidence of several noncommunicable diseases, including cardiovascular disease, type 2 diabetes, breast cancer, and colon cancer [12]. It can also help prevent cognitive decline in older adults and promote healthy aging [13, 14]. Moreover, different types of physical activity have varying effects on health. A prospective cohort study of 104,046 adults found that while higher leisure-time physical activity was associated with reduced risks of major adverse cardiovascular events (MACE) and all-cause mortality, higher occupational physical activity was associated with increased risks [15].

Research directly examining physical activity (PA) and biological aging remains relatively limited. A cross-sectional study in Japan showed that PA measured by accelerometers was associated with slower epigenetic aging, whereas self-reported PA showed no association with epigenetic aging [16]. Ting Yu Lu et al. reported that PA was associated with greater longevity and reduced accelerated biological aging in a follow-up survey of the GBCS, and suggested that the protective effect of PA against accelerated aging was partly mediated by lipids [17]. Phenotypic Age Acceleration (PhenoAgeAccel), defined as the residual of phenotypic age regressed on chronological age, reflects the degree to which an individual’s biological age deviates from chronological age. Another study based on a U.S. population sample found that individuals who engaged in physical activity, regardless of whether they met the guideline, had significantly lower PhenoAgeAccel scores than those without leisure-time physical activity (LTPA) (P < 0.05) [18]. However, most existing studies have focused on single PA domains or have not distinguished between domains, and no research has systematically assessed the optimal doses or combined effects of PA across domains in relation to delayed aging. These limitations may partly explain why current conclusions regarding PA and delayed aging remain inconsistent.

To address these questions, we used nationally representative data from the National Health and Nutrition Examination Survey (NHANES). We estimated biological age using the Klemera–Doubal Method (KDM-BA), Phenotypic Age (PhenoAge), and Homeostatic Dysregulation (HD), examined the associations between various domains of physical activity and delayed biological aging, and analyzed their combined effects to identify the optimal durations of activity across domains.

## MATERIALS AND METHODS

### Data Source

This study analyzed cross-sectional data from four NHANES survey cycles conducted during 2007–2010 and 2015–2018. NHANES is a continuous, nationally representative survey conducted biennially in the United States, employing a complex, multistage, stratified probability sampling design to select participants from the civilian, non-institutionalized population. Data are collected through structured household interviews and standardized health examinations in Mobile Examination Centers, which include physical measurements and laboratory tests for participants of all ages, thereby ensuring highquality and nationally representative health information. The NHANES protocols were approved by the Ethics Review Board of the National Center for Health Statistics (NCHS ERB), and all participants provided written informed consent. Detailed descriptions of the survey design, data collection procedures, and publicly available datasets are available at the NHANES website (https://www.cdc.gov/nchs/nhanes/index.html).

From the NHANES datasets spanning 1999–2023, we restricted our analysis to survey cycles conducted in 2007–2010 and 2015–2018, because the 1999–2006 and 2019–2023 cycles did not employ the standard GPAQ questionnaire for physical activity assessment, and the 2011–2014 cycles lacked data on Creactive protein (CRP). This restriction yielded 39,911 participants. We then applied the following exclusion criteria step by step: participants younger than 20 years (n = 16,471), pregnant women (n = 250), those missing physical activity data (n = 242), education or marital information (n = 46), and biomarker data (n = 4,540). After these exclusions, the final analytic sample comprised 18,362 adults with complete information on PA, biomarkers, and covariates.

### Measures

#### Physical Activity

In NHANES, physical activity is assessed using the self-reported Global Physical Activity Questionnaire (GPAQ), which captures the time participants spend in vigorous- and moderate-intensity activities across three domains: occupational (OPA), transportation (TPA), and leisure-time (LTPA). OPA includes household chores and manual labor such as carrying or lifting heavy objects, digging, or construction work. TPA refers to active transportation, including walking or cycling to school, work, or shopping. LTPA encompasses purposeful exercise and recreational activities, such as sports (e.g., basketball, swimming), cycling, and brisk walking. For each reported activity, participants are further asked about the duration of a typical session and the frequency per week.

To quantify physical activity, total weekly time and energy expenditure for each domain were calculated according to the NHANES physical activity and GPAQ analysis guidelines [19]. Total physical activity volume was calculated as minutes of moderate-intensity activity plus twice the minutes of vigorous-intensity activity. Vigorous, moderate, and transportation physical activities were assigned values of 8, 4, and 4 Metabolic Equivalents (METs), respectively. Finally, total weekly physical activity was categorized as: none activity (0 minutes, 0 METs), Low (1–149 minutes, 1–600 METs), Middle (150–300 minutes, 600–1200 METs), and High (> 300 minutes, > 1200 METs).

#### Biological Age Prediction

In this study, biological aging was assessed using three validated indicators: the Klemera–Doubal Method Biological Age (KDMBA) [20], the Phenotypic Age (PhenoAge) algorithm [21], and the Homeostatic Dysregulation (HD) index. KDM-BA and PhenoAge provide direct estimates of biological age, whereas HD, although not a direct age estimator, reflects preclinical dysregulation along the health–disease continuum and has been shown to predict morbidity and mortality risk [22–24].

Following previous studies [25, 26], we selected specific clinical and hematological biomarkers for these measures. KDM-BA was derived from systolic blood pressure, albumin, alkaline phosphatase, blood urea nitrogen, creatinine, C-reactive protein, glycosylated hemoglobin, and total cholesterol. PhenoAge was calculated using chronological age together with nine biomarkers (albumin, alkaline phosphatase, creatinine, glucose, C-reactive protein, lymphocyte percentage, mean cell volume, red cell distribution width, and white blood cell count). HD was computed using the same biomarkers as PhenoAge, excluding chronological age. All measures were implemented using the “BioAge” package in R (https://github.com/dayoonkwon/BioAge).

To define biological aging status, we classified participants as having delayed aging if KDM-BA or PhenoAge acceleration was < 0, or if HD values were below the sample median, consistent with prior literature [26].

#### Covariates

Covariates were obtained from standardized NHANES questionnaires. Demographic variables included age (continuous), sex (male or female), race/ethnicity (Mexican American, Non-Hispanic White, Non-Hispanic Black, other Hispanic, and other groups), educational level (less than high school, high school, more than high school), marital status (unmarried, married, divorced), and poverty index (continuous). Lifestyle variables included smoking, drinking, sleep duration, and sedentary behavior. Chronic disease status was determined from participants’ self-reported history of diabetes, cardiovascular disease (CVD), or cancer. For categorical variables, missing data were coded as missing, whereas for continuous variables, multiple imputation was performed using the mice package in R.

### Statistical Analysis

Baseline characteristics were summarized as mean ± SE for continuous variables and as frequency (n) and percentage (%) for categorical variables. Differences across physical activity domains were assessed using ANOVA or Kruskal–Wallis tests for continuous variables and Chi-square tests for categorical variables. Multivariable logistic regression models were used to estimate odds ratios (ORs) and 95% confidence intervals (CIs) for the associations between physical activity and delayed biological aging. Dose–response relationships were assessed with restricted cubic spline regression, placing knots at the 25^th^, 50^th^, and 75^th^ percentiles. To formally evaluate heterogeneity and interaction effects, we included cross-product terms and applied likelihood ratio tests to determine whether combined effects of physical activity across domains exceeded the expected cumulative effects on additive and multiplicative scales.

To address potential residual confounding by socioeconomic status (SES), we constructed composite SES classes using latent class analysis (LCA). Four observed SES indicators—educational attainment, household income (PIR or income categories), occupational category, and medical insurance coverage—were harmonized into three ordered levels (low, medium, high). A series of LCA models was fitted, and both 3-class (Low / Medium / High) and 4-class (Low / Medium-Low / Medium-High / High) solutions were retained based on information criteria (AIC, BIC), entropy, parsimony, and interpretability. Class assignment was determined by maximum posterior probability. We then (1) adjusted for the LCA-derived SES classes as covariates (Table S17) and (2) performed SES-stratified analyses under both the 3-class (Table S18) and 4-class (Table S19) solutions. As a robustness check, we compared estimates with and without LCA-SES adjustment to evaluate potential attenuation.

All analyses were conducted using R software (version 4.3.3) in November 2024. A two-sided P < 0.05 was considered statistically significant for descriptive analyses. To account for multiple testing across nine primary comparisons (three biological aging outcomes × three exposure contrasts), Bonferroni correction was applied, yielding an adjusted significance threshold of P < 0.0056.

## RESULTS

### Baseline characteristics

The final analytic sample comprised 18,362 participants. Significant differences in age, sex, race/ethnicity, marital status, educational level, and poverty index ratio (PIR) were observed across physical activity groups (P < 0.05). Younger individuals were more likely to engage in transportation physical activity (TPA) and leisure-time physical activity (LTPA). Across all three domains, the proportion of males consistently exceeded that of females. Females, individuals with a higher PIR, non-smokers, alcohol consumers, and those with higher educational attainment were more likely to engage in LTPA. The prevalence of delayed aging was highest among participants engaging in LTPA. Conversely, participants engaged in occupational physical activity (OPA) had the highest prevalence of cardiovascular disease (CVD), cancer, and diabetes, and the lowest likelihood of delayed aging (Table 1).

**TABLE 1:** Participant characteristics stratified by different physical activity

	Overall (18362)	OPA^a^ (7941)	TPA^a^ (4336)	LTPA^a^ (8616)	P-Value^b^
**Age(year)**	50.2 (17.7)	47.1 (17.1)	46.2 (17.4)	46.7 (17.4)	0.013

**PIR**	2.5 (1.6)	2.5 (1.6)	2.2 (1.6)	2.9 (1.7)	0.001

**Sex**					0.001
Male	9095 (49.5)	4576 (57.6)	2344 (54.1)	4538 (52.7)	
Female	9267 (50.5)	3365 (42.4)	1992 (45.9)	4078 (47.3)	

**Race**					0.001
Mexican American	3118 (17.0)	1291 (16.3)	819 (18.9)	1246 (14.5)	
Other Hispanic	2019 (11.0)	758 (9.5)	547 (12.6)	839 (9.7)	
Non-Hispanic White	7841 (42.7)	3847 (48.4)	1525 (35.2)	3919 (45.5)	
Non-Hispanic Black	3452 (18.8)	1401 (17.6)	861 (19.9)	1584 (18.4)	
Other Race	1932 (10.5)	644 (8.1)	584 (13.5)	1028 (11.9)	

**Education**					0.001
Below high school	4669 (25.4)	1725 (21.7)	1262 (29.1)	1323 (15.4)	
High school	4252 (23.2)	2083 (26.2)	904 (20.8)	1775 (20.6)	
College or above	9441 (51.4)	4133 (52.0)	2170 (50.0)	5518 (64.0)	

**Marital**					0.001
Never married	3154 (17.2)	1467 (18.5)	1097 (25.3)	1786 (20.7)	
Married/living with partner	11162 (60.8)	5023 (63.3)	2382 (54.9)	5251 (60.9)	
Widowed/divorced	4046 (22.0)	1451 (18.3)	857 (19.8)	1579 (18.3)	

**BMI, kg/m^2^**					0.001
≤ 25	5099 (27.8)	2166 (27.3)	1472 (33.9)	2685 (31.2)	
> 25 to < 30	6140 (33.4)	2595 (32.7)	1454 (33.5)	3003 (34.9)	
≥ 30	7123 (38.8)	3180 (40.0)	1410 (32.5)	2928 (34.0)	

**Sleep**					0.001
< 6	2357 (12.8)	1146 (14.4)	522 (12.0)	928 (10.8)	
≥ 6 to < 7	3575 (19.5)	1625 (20.5)	850 (19.6)	1675 (19.4)	
≥ 7 to < 8	4926 (26.8)	2195 (27.6)	1137 (26.2)	2549 (29.6)	
≥ 8	7504 (40.9)	2975 (37.5)	1827 (42.1)	3464 (40.2)	

**Sedentary Behavior^c^**					0.001
Q1	5908 (32.2)	3017 (38.0)	1698 (39.2)	2644 (30.7)	
Q2	4575 (24.9)	2303 (29.0)	1083 (25.0)	2228 (25.9)	
Q3	4727 (25.7)	1803 (22.7)	990 (22.8)	2216 (25.7)	
Q4	3152 (17.2)	818 (10.3)	565 (13.0)	1528 (17.7)	

**Smoking**					0.001
Ever smoker	8245 (44.9)	3899 (49.1)	1925 (44.4)	3526 (40.9)	
Never smoker	10117 (55.1)	4042 (50.9)	2411 (55.6)	5090 (59.1)	

**Drinking**					0.001
Ever	11248 (61.3)	5286 (66.6)	2724 (62.8)	5775 (67.0)	
Never	5947 (32.4)	2240 (28.2)	1316 (30.4)	2341 (27.2)	
Missing (%)	1167 (6.4)	415 (5.2)	296 (6.8)	500 (5.8)	

**Diabetes**					0.143
Yes	2490 (13.6)	832 (10.5)	448 (10.3)	827 (9.6)	
No	15872 (86.4)	7109 (89.5)	3888 (89.7)	7789 (90.4)	

**Cancer**					0.001
Yes	1767 (9.6)	729 (9.2)	278 (6.4)	757 (8.8)	
No	16578 (90.3)	7204 (90.7)	4054 (93.5)	7854 (91.2)	
Missing (%)	17 (0.1)	8 (0.1)	4 (0.1)	5 (0.1)	

**CVD**					0.002
Yes	1984 (10.8)	695 (8.8)	311 (7.2)	652 (7.6)	
No	16378 (89.2)	7246 (91.2)	4025 (92.8)	7964 (92.4)	

**KDM-BA**	48.2 (17.3)	45.4 (16.3)	44.3 (16.8)	44.6 (16.5)	0.001

**KDM-BA delay aging^d^**	12102 (65.9)	5083 (64.0)	2871 (66.2)	5760 (66.9)	0.001

**PhenoAge**	47.6 (19.2)	44.6 (18.3)	43.1 (18.5)	43.5 (18.4)	0.001

**PhenoAge delay aging^d^**	13452 (73.3)	5837 (73.5)	3309 (76.3)	6757 (78.4)	0.001

**HD**	1.5 (1.0)	1.4 (0.9)	1.4 (0.9)	1.4 (0.9)	0.001

**HD delay aging^e^**	9181 (50.0)	4259 (53.6)	2328 (53.7)	4878 (56.6)	0.001

a Occupational physical activity (OPA), transportation physical activity (TPA), and leisure-time physical activity (LTPA) refer to participants who engaged in the corresponding types of physical activity.

b The characteristics of people who participated in different types of physical activities were examined using ANOVA or Kruskal-Wallis tests for continuous variables and Chi-square tests for categorical variables.

c Sedentary behavior is divided into quartiles according to the length of time spent sitting each day.

d Klemera-Doubal Method (KDM-BA) and Phenotypic Age (Phenoage) delay aging are both defined as biological age being less than actual age, that is, the residual is less than 0.

e For Homeostatic Dysregulation (HD), below the median is defined as decelerated aging.

### Associations and Dose-Response Relationships between Physical Activity Domains, Intensity Levels, and Delaying Biological Aging

After adjusting for age, sex, race/ethnicity, educational attainment, marital status, poverty index ratio (PIR), smoking, alcohol consumption, sleep duration, sedentary behavior, cardiovascular disease, diabetes, and cancer, we assessed associations between occupational, transportation, and leisure-time physical activity and delayed biological aging. Results were evaluated using Bonferroni correction (P < 0.0056).

**FIG. 1:**
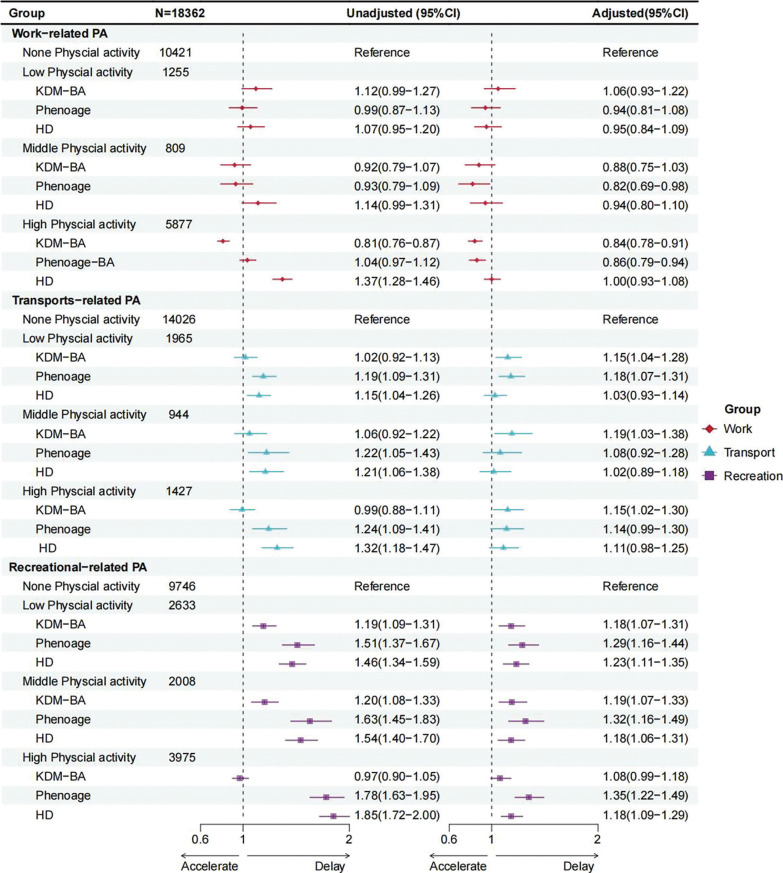
Forest plot of odds ratios and 95% confidence intervals for different domains and levels of physical activity in delaying aging ^**a**^None Physical activity (0 minutes/week), Low Physical activity (1–149 minutes/week), Middle Physical activity (150–300 minutes/ week), and High Physical activity (over 300 minutes/week). ^**b**^Adjusted models adjusted for covariates age, sex, education level, marital status, PIR, race, smoking, drinking, sleep, sedentary behavior, diabetes, CVD, and cancer.

For occupational physical activity (OPA), middle levels were associated with lower odds of delayed aging in PhenoAge (OR = 0.82, 95% CI = 0.69–0.98), but this association was not significant after correction. High OPA was associated with lower odds of delayed aging in both KDM-BA (OR = 0.84, 95% CI = 0.78–0.91) and PhenoAge (OR = 0.86, 95% CI = 0.79–0.94), and these associations remained significant, while low OPA showed no significant association (Table S1–3, Figure 1). For transportation physical activity (TPA), low (KDM-BA, OR = 1.15; PhenoAge, OR = 1.18), middle (KDM-BA, OR = 1.19), and high (KDM-BA, OR = 1.15) levels were initially associated with higher odds of delayed aging, but none remained significant after correction. For leisure-time physical activity (LTPA), both low (KDM-BA, OR = 1.18; PhenoAge, OR = 1.29; HD, OR = 1.23) and middle (KDM-BA, OR = 1.19; PhenoAge, OR = 1.32; HD, OR = 1.18) levels were consistently associated with delayed aging across all three measures, and these associations remained significant. High LTPA was also associated with delayed aging in PhenoAge (OR = 1.35, 95% CI = 1.22–1.49) and HD (OR = 1.18, 95% CI = 1.09–1.29), but not in KDM-BA (OR = 1.08, 95% CI = 0.99–1.18). Moreover, in PhenoAge, the ORs increased progressively with higher levels of LTPA (Table S1–3, Figure 1).

When stratified by age, differences in OPA were observed between participants aged < 65 and those ≥ 65 years. In younger adults (< 65 years), OPA was associated with lower odds of delayed aging (KDM, OR = 0.74, 95% CI = 0.68–0.80, P < 0.001; PhenoAge, OR = 0.84, 95% CI = 0.76–0.92, P < 0.001), whereas no significant association was observed in older adults (≥ 65 years) (Table S4) In stratified analyses by chronic disease status, higher levels of TPA and LTPA were more strongly associated with delayed aging among participants with chronic diseases, whereas higher levels of OPA were inversely associated with delayed aging among those without chronic diseases (Table S5).

**FIG. 2:**
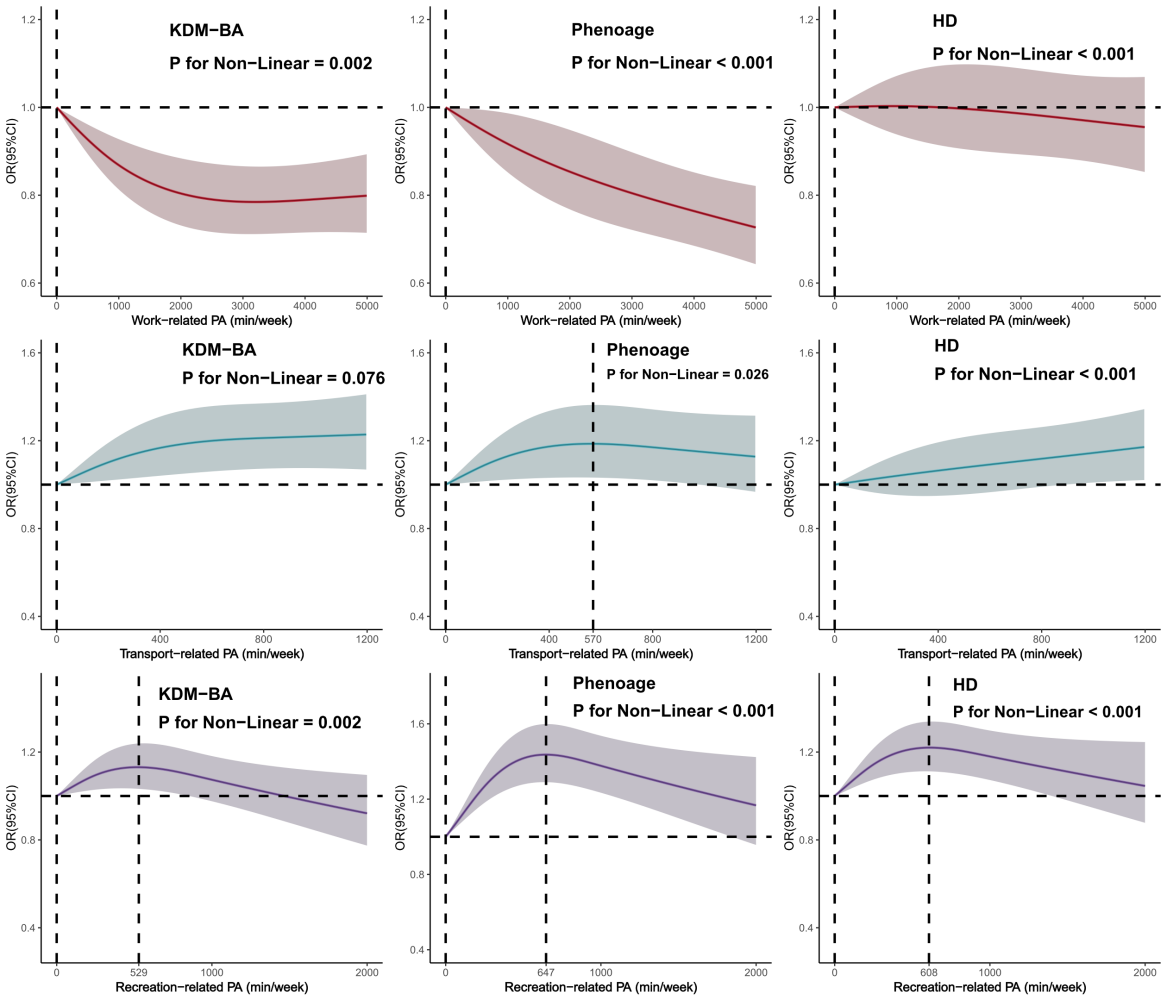
Dose-response relationship between different types of physical activity and KDM-BA, Phenoage, and HD in delaying aging ^**a**^Dose-response relationships for all types of physical activity were adjusted for the covariates age, sex, education level, marital status, PIR, ethnicity, smoking, drinking, sleep, sedentary behavior, diabetes, CVD, and cancer.

Subsequently, we used restricted cubic spline functions to evaluate the dose-response relationships between different types of physical activity and the probability of delayed aging for the three biological aging indicators. After multivariate adjustment, in the OPA domain, low, middle, and high OPA levels were all associated with a lower likelihood of delayed aging; and as the level of physical activity increased, the proportion of delayed aging further decreased. Compared with OPA, TPA was only associated with an increased proportion of delayed aging, regardless of whether activity levels were low, middle, or high. Furthermore, as the level of physical activity increased, the proportion of delayed aging increased, reaching a plateau at approximately 570 minutes per week, beyond which it did not increase further. For LTPA, the dose–response relationship exhibited an inverted U-shape (KDM-BA, PhenoAge, HD), with the maximum effects observed at approximately 529, 647, and 608 minutes of activity per week, respectively. However, once the duration of activity exceeded these peak levels, the effect of delaying aging gradually weakened (Figure 2).

### Joint Associations between Physical Activity Types and Delaying Biological Aging

**FIG. 3:**
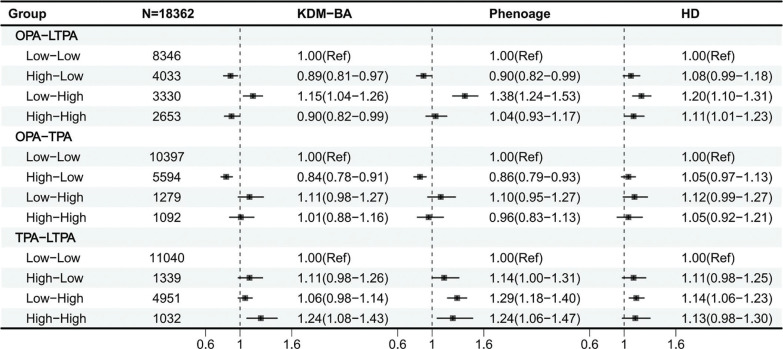
Joint associations between different types of physical activity and delaying biological aging (forest plot) ^**a**^Physical activity levels were categorized as greater than or equal to 150 minutes per week or less than 150 minutes per week. ^**b**^All analyses of joint effects were adjusted for covariates including age, sex, education level, marital status, PIR, race, smoking, drinking, sleep, sedentary behavior, diabetes, CVD, and cancer.

Multivariable logistic regression analysis showed that middle and high levels of OPA were associated with lower odds of delayed aging. For the joint association analysis, none physical activity and lowlevel activity were combined into a low-level category (0–149 minutes, 0–600 METs), while middle and high levels were grouped into a high-level category (≥ 150 minutes, ≥ 600 METs). We then examined the joint associations of the three domains across these two levels (Table 2, Figure 3).

**TABLE 2:** Joint associations between different types of physical activity and delaying biological aging

			N = 18362	KDM-BA OR (95%CI)	P^b^	Phenoage OR (95%CI)	P^b^	HD OR (95%CI)	P^b^
**OPA^a^-LTPA^a^**	Low OPA	Low LTPA	8346	1.00 (Ref)		1.00 (Ref)		1.00 (Ref)
	High OPA	Low LTPA	4033	0.89 (0.81–0.97)	**0.005**	0.90 (0.82–0.99)	0.025	1.08 (0.99–1.18)	0.072
	High LTPA	Low OPA	3330	1.15 (1.04–1.26)	**0.004**	1.38 (1.24–1.53)	**0.001**	1.20 (1.10–1.31)	**0.001**
	High LTPA	High OPA	2653	0.90 (0.82–0.99)	0.041	1.04 (0.93–1.17)	0.504	1.11 (1.01–1.23)	0.033

**OPA^a^-TPA^a^**	Low OPA	Low TPA	10397	1.00 (Ref)		1.00 (Ref)		1.00 (Ref)
	High OPA	Low TPA	5594	0.84 (0.78–0.91)	**0.001**	0.86 (0.79–0.93)	**0.001**	1.05 (0.97–1.13)	0.235
	High TPA	Low OPA	1279	1.11 (0.98–1.27)	0.105	1.10 (0.95–1.27)	0.204	1.12 (0.99–1.27)	0.084
	High TPA	High OPA	1092	1.01 (0.88–1.16)	0.894	0.96 (0.83–1.13)	0.632	1.05 (0.92–1.21)	0.447

**TPA^a^-LTPA^a^**	Low TPA	Low LTPA	11040	1.00 (Ref)		1.00 (Ref)		1.00 (Ref)
	High TPA	Low LTPA	1339	1.11 (0.98–1.26)	0.108	1.14 (1.00–1.31)	0.062	1.11 (0.98–1.25)	0.106
	High LTPA	Low TPA	4951	1.06 (0.98–1.14)	0.145	1.29 (1.18–1.40)	**0.001**	1.14 (1.06–1.23)	**0.001**
	High LTPA	High TPA	1032	1.24 (1.08–1.43)	**0.003**	1.24 (1.06–1.47)	0.008	1.13 (0.98–1.30)	0.087

a Physical activity levels were categorized as greater than or equal to 150 minutes per week or less than 150 minutes per week.

b All analyses of joint effects were adjusted for covariates including age, sex, education level, marital status, PIR, race, smoking, drinking, sleep, sedentary behavior, diabetes, CVD, and cancer.

c IP values that remained significant after Bonferroni correction (P < 0.0056) are shown in bold.

Among OPA combinations, low OPA combined with high LTPA or TPA was associated with a higher likelihood of delayed aging (OR > 1). In contrast, high OPA combinations showed neutral to adverse associations with biological aging, regardless of TPA or LTPA level, compared with low OPA combinations. Moreover, all TPA–LTPA combinations were beneficial for delaying aging (OR > 1) (Table S6–15). Interaction effects between activity types were further analyzed using KDM-BA, PhenoAge, and HD. Both additive (KDM-BA, RERI = –0.129, 95% CI = –0.213 to –0.044) and multiplicative (PhenoAge, OR = 0.849, 95% CI = 0.725–0.995, P = 0.043) interactions were observed between OPA and LTPA. Additive interactions were also observed between TPA and LTPA (KDM-BA, RERI = 0.277, 95% CI = 0.053–0.500; PhenoAge, RERI = 0.383, 95% CI = 0.051–0.715). No additive or multiplicative interactions were detected between OPA and TPA in KDM-BA, PhenoAge, or HD (Table S16).

### Sensitivity analyses

Individually, middle to high levels of OPA were associated with lower odds of delayed aging (OR < 1), whereas TPA and LTPA, at all levels, were associated with delayed aging (OR > 1, (Table S1–5)). In sensitivity analyses of combined associations, high OPA combined with low LTPA was associated with lower odds of delayed aging compared with low OPA and low LTPA. Conversely, high LTPA combined with low OPA was associated with higher odds of delayed aging.

Furthermore, to test the robustness of the HD-based results, we redefined delayed aging using alternative thresholds (65^th^ and 75^th^ percentiles), which were chosen to approximate the proportions of delayed aging observed when using KDM-BA and PhenoAge. We also modeled HD as a continuous outcome. These sensitivity analyses yielded results consistent with our main findings, supporting the robustness of the observed associations (Table S2).

After additionally adjusting for LCA-derived SES (3- and 4-class), the inverse association between high OPA and delayed aging remained essentially unchanged (Table S17): KDM-BA OR 0.84 (0.78–0.90), P < 0.001; PhenoAge OR 0.82 (0.76–0.89), P < 0.001; HD OR 1.01 (0.94–1.08), P = 0.83. In SES-stratified analyses (Table S18–S19), the inverse association persisted across strata, with Low SES (3-class) showing KDM-BA OR 0.79 (0.71–0.87), P < 0.001 and PhenoAge OR 0.83 (0.74–0.93), P < 0.001; and Medium-High / High SES (4-class) showing KDM-BA OR 0.73 (0.65–0.82), P < 0.001 and Phenoage OR 0.79 (0.67–0.95), P = 0.010, respectively; PhenoAge OR 0.76 (0.66–0.88), P < 0.001 and 0.80 (0.66–0.96), P = 0.017. HD remained null across strata. These findings suggest that enhanced SES control and stratification do not materially alter the core OPA associations, while highlighting SES-related heterogeneity in effect sizes.

## DISCUSSION

This study highlights that different types of physical activity differ in their associations with biological aging. Excessive OPA may have the opposite effect, being associated with a reduced likelihood of delayed aging. In contrast, LTPA was significantly associated with an increased likelihood of delayed aging (OR > 1). Transportation physical activity (TPA) generally increased the likelihood of delayed aging, though subtle differences were observed across aging indicators. In the GPAQ questionnaire, TPA refers to walking and cycling, which resemble LTPA and may therefore confer similar health benefits [18]. In combined analyses, high OPA was associated with a lower likelihood of delayed aging compared with the combination of low OPA and low LTPA. Similarly, in the OPA–TPA group, results mirrored those of the OPA–LTPA group, suggesting that high OPA was adversely associated with delayed aging. To our knowledge, this is the first study to comprehensively evaluate the joint effects of physical activity across domains on biological aging. Additionally, we employed three widely used biological aging indicators to strengthen the validity of our conclusions.

In this study, we applied the Klemera–Doubal Method (KDM-BA) and the PhenoAge algorithm to estimate participants’ biological age. Both methods have been validated in multi-ethnic elderly populations and shown to predict disease, disability, and mortality [27, 28]. However, controversy remains because both algorithms incorporate chronological age into their calculations [29, 30]. To address this, we also calculated Homeostatic Dysregulation (HD) as an alternative measure of aging. Cohen et al. developed HD as a single measure by quantifying deviations of multiple clinical biomarkers from defined “physiological standards” or baseline states [23]. By employing multiple aging indices, this study enhances the robustness of its findings. Nonetheless, in some results, HD was inconsistent with KDM-BA and PhenoAge, likely reflecting the different emphases of these indices. KDM-BA and PhenoAge primarily focus on predicting age-related disease risk [31], whereas HD emphasizes deviations from healthy reference samples [23]. Another possible explanation is the absence of standardized reference values for HD, which complicates accurate dichotomization. In this study, we used the median value method; future research could validate these findings using additional aging indices.

The 2020 WHO physical activity guidelines emphasize that adults should regularly engage in physical activity to achieve health benefits, irrespective of domain, as evidence has not clearly differentiated health effects across activity domains [11]. However, emerging evidence indicates that variations in domain and dose of physical activity may lead to markedly different health outcomes. A review on physical activity and worker health reported that leisure-time activity benefits all workers, whereas high levels of occupational activity are associated with increased risks of all-cause mortality, cardiovascular disease, and diabetes compared with lower levels of work-related activity [32]. A 24-year cohort study similarly found that, compared with inactive OPA, light OPA was associated with increased overall mortality, and heavy OPA with increased cardiovascular disease mortality [33]. A UK Biobank study of ~284,000 participants found that leisure and transportation activities exert potential protective effects against aging, with greater adherence associated with lower aging indices (LTL deviation and BAA) [34]. Consistent with prior literature, we found that low, middle, and high levels of LTPA were generally associated with a higher likelihood of delayed aging across all three biological aging measures. Eight of nine associations were statistically significant (OR > 1, P < 0.001), with the only exception being highlevel LTPA and KDM-BA (P = 0.081). In contrast, excessive OPA was associated with the opposite effect (S16).

This study found an association between OPA and a lower rate of delayed aging, and possible explanations for these results include work-related psychological stress in high-stress or high-demand working conditions [35, 36], unhealthy lifestyles [37], and adverse biological changes [38]. In addition, Andreas Holtermann et al. summarized the reasons for this physical activity paradox [39]. Specifically, work-related physical activity is often of low intensity and long duration, which fails to effectively stimulate improvements in cardiopulmonary function, tends to cause excessive fatigue, and may not allow sufficient recovery. In addition, work-related physical activity often increases blood pressure and inflammation levels, and exposure to environmental toxins in some workplaces may also be the cause of this contradiction. Another point worth noting is that people who participate in work-related physical activity often have lower income levels, and lower socioeconomic status is often associated with less healthy physical conditions [40].

Because SES is a fundamental determinant of both occupational context and health, we derived composite SES classes using LCA to capture multi-dimensional socioeconomic heterogeneity (education, income, occupation, and medical insurance; each in three ordered levels). Adjusting for the LCA-derived SES (3- and 4-class) did not attenuate the inverse association between high OPA and delayed biological aging (e.g., PhenoAge OR 0.86 to 0.82 after SES adjustment), and SES-stratified analyses further showed consistent inverse associations across strata (Table S17–S19). These results argue against SES being the sole explanation for the observed ‘physical activity paradox’ and suggest that work-related biomechanical load, insufficient recovery, psychosocial stress, and adverse work environments may contribute to aging-related dysregulation beyond socioeconomic differences. Notably, the lack of association with HD across models aligns with prior work indicating that OPA’s adverse features are more readily captured by aging algorithms tied to morbidity/mortality risk (e.g., KDM-BA, PhenoAge), whereas HD emphasizes deviation from a healthy reference rather than risk prediction.

While LCA improves SES control by integrating multiple indicators, residual confounding cannot be completely ruled out, including unmeasured aspects of job insecurity, toxic exposures, or informal labor conditions. Nevertheless, the stability of estimates across SESadjusted and SES-stratified models increases confidence in the robustness of our findings. Collectively, these findings reinforce that the detrimental effects of occupational physical activity on biological aging cannot be solely attributed to socioeconomic disadvantage but may involve complex interactions between physical strain and recovery imbalance within specific work environments.

In this study, we found that TPA and LTPA were associated with a higher likelihood of delayed aging. There are several possible biological pathways through which TPA and LTPA may delay aging. First, regular aerobic exercise is associated with maintaining telomere length [41–44], enhancing mitochondrial biogenesis through activation of PGC-1α [45], and reducing levels of proinflammatory cytokines, thereby counteracting the chronic low-grade inflammation characteristic of aging [8, 46]. Second, physical activity can improve insulin sensitivity and metabolic health [47] and increase circulating levels of brain-derived neurotrophic factor (BDNF), potentially protecting against cognitive decline [48, 49]. Finally, LTPA can improve mental health and alleviate psychological stress [50]. Previous studies have shown that stress may be a key factor affecting aging, and reducing stress may again change biological age [8, 36]. A previous study found that 25-hydroxyvitamin D [25(OH)D] and LTPA have a joint effect on delaying biological aging. Compared to the group lacking vitamin D and physical activity, the group with sufficient vitamin D and adequate physical activity had an OR of PhenoAgeAccel of 0.657 [0.549, 0.787] (P < 0.001), indicating a 34.3% reduction in the probability of accelerated aging [51]. These mechanisms provide a plausible biological basis to support the associations observed in our study.

Beyond statistical significance, the magnitude of these associations also carries important public health implications. Although the observed effect sizes were modest (ORs ranging from approximately 0.84 to 1.35), they should be interpreted in the context of population-level impact. Even small individual-level changes in biological aging can translate into substantial differences in morbidity, disability, and mortality risk when aggregated across populations [31]. For instance, an OR of 1.35 for leisure-time physical activity corresponds to roughly a 35% higher probability of maintaining a younger biological age. Although direct translation into “years of biological aging” is not available, prior studies have reported that similar lifestyle-related differences in biological aging indices typically correspond to approximately 1–2 years of difference between exposure groups [7, 31, 52]. These magnitudes, though modest, are comparable to effects observed in lifestyle–aging studies [7, 53] and diet pattern–aging associations [52]. Moreover, evidence from epigenetic studies indicates that age acceleration metrics predict healthy longevity [54], underscoring the clinical relevance of even modest shifts. Therefore, while statistically modest, the observed associations are likely biologically meaningful and relevant for public health strategies promoting active lifestyles and healthy aging.

Sensitivity analyses suggested differences by age group and chronic disease status. A significant association between OPA and a lower likelihood of delayed aging was observed only among participants < 65 years, but not among those ≥ 65 years (Table S4). Several explanations are possible. First, because NHANES combines workrelated and housework-related activities, OPA in younger adults may primarily reflect high-intensity occupational labor (e.g., cargo handling), whereas in older adults it may reflect moderate- to low-intensity housework (e.g., cleaning, sweeping). Second, a health selection effect may exist: older adults who continue OPA beyond age 65 are likely healthier, while those with greater chronic disease burden may exit the labor market earlier. Finally, the types of jobs held by older adults may differ, often involving lower-intensity activities (e.g., tour guiding with walking), which impose substantially less physical load than heavy labor. We also observed that the benefits of LTPA for delaying aging were greater among individuals with chronic diseases. This may be because such individuals are generally in poorer health and less likely to engage in LTPA, so those who do participate may derive greater benefits. Accordingly, the WHO recommends that adults with chronic diseases engage in moderate- to high-intensity aerobic physical activity each week, in line with guideline requirements.

Although physical activities in different domains have been shown to delay biological aging and reduce the risk of specific chronic diseases, their joint effects on physical and mental health remain unclear. In this study, we examined the joint effects of OPA, TPA, and LTPA on biological aging. Our analysis indicated that the potential adverse effects of OPA on delayed aging may be partially offset by beneficial LTPA, while combined TPA and LTPA conferred even greater benefits.

This study has several notable strengths. First, we utilized nationally representative cross-sectional data to examine associations between different domains and levels of physical activity and delayed biological aging, and validated findings across three biological aging indicators. Second, we were the first to investigate the joint effects of physical activity across domains on biological aging. Third, to enhance the robustness of our results, we applied Bonferroni correction to account for multiple testing across nine primary comparisons, thereby reducing the likelihood of false-positive findings.

However, this study has several limitations. Firstly, the cross-sectional study design restricts the analysis to associations observed at a single time point. Therefore, we cannot determine the causal relationship between these physical activities and biological aging. Secondly, the physical activity data is based on self-reports from participants, which may involve subjective judgments, recall biases, and potential social desirability bias. Thirdly, the classification of physical activity across different domains (e.g., occupational, transportation, and leisure-time) may be subject to non-differential misclassification. Such misclassification could bias domain-specific effect estimates toward the null and may also complicate the interpretation of interaction analyses. Fourthly, the absence of a universally accepted gold standard for assessing biological aging may limit the generalizability of the findings, and in particular, the operationalization of delayed aging for the HD relied on distribution-based thresholds without a clear biological benchmark, which may further affect comparability across studies. Fifthly, while the application of Bonferroni correction strengthens the credibility of significant associations, it is also highly conservative and may increase the risk of false negatives, potentially obscuring meaningful associations. Finally, this study only discussed the duration of different types of physical activities and did not address their intensity, which should be explored in future research.

## CONCLUSIONS

This study found that high OPA was significantly associated with a lower likelihood of delaying aging (KDM-BA, OR = 0.84, 95% CI = 0.78–0.91, P < 0.001; PhenoAge, OR = 0.86, 95% CI = 0.79–0.94). Analysis of the combined effects of different physical activities on delayed aging showed that, compared with the combination of low OPA and low LTPA, increased OPA levels were associated with a lower probability of delayed aging, while increased LTPA levels were associated with a higher probability of delayed aging. These research results highlight the different roles that physical activities in different fields and at different levels play in delaying biological aging, which is of great significance to public health strategies aimed at delaying aging.

## Supplementary Material

Associations of physical activity domains and delayed biological aging: assessing heterogeneity and interaction of effects

## Data Availability

The dataset(s) supporting the conclusions of this article is(are) available in the NHANES website (https://wwwn.cdc.gov/nchs/nhanes/).
