# Supplementary material for: Associations of physical activity domains and delayed biological aging: assessing heterogeneity and interaction of effects
Source: Biol Sport. 2026 Feb 6;43:847–59. doi: 10.5114/biolsport.2026.158670 (PMC13217144; doi:10.5114/biolsport.2026.158670)
Supplement: Associations of physical activity domains and delayed biological aging: assessing heterogeneity and interaction of effects [file JBS-43-57444-s1.pdf]

TABLE S1. Odds Ratios and confidence intervals of different types of physical activities in delaying aging[b]

	N = 18362	Model1[b]	Model2[b]	Model3[b]	Model4[b]	Model5[b]
**Work-related PA**						
None Physical activity[a]	10421	1.00 (Ref)	1.00 (Ref)	1.00 (Ref)	1.00 (Ref)	1.00 (Ref)
Low Physical activity[a]	1255					
KDM-BA		1.12 (0.99–1.27)	1.09 (0.96–1.25)	1.08 (0.94–1.23)	1.07 (0.94–1.22)	1.06 (0.93–1.22)
Phenoage		0.99 (0.87–1.13)	0.99 (0.87–1.14)	0.97 (0.85–1.12)	0.95 (0.82–1.09)	0.94 (0.81–1.08)
HD		1.07 (0.95–1.20)	1.00 (0.87–1.14)	0.98 (0.87–1.11)	0.96 (0.85–1.09)	0.95 (0.84–1.09)
Middle Physical activity[a]	809					
KDM-BA		0.92 (0.79–1.07)	0.92 (0.79–1.08)	0.91 (0.78–1.06)	0.89 (0.76–1.04)	0.88 (0.75–1.03)
Phenoage		0.93 (0.79–1.09)	0.91 (0.77–1.07)	0.88 (0.75–1.04)	0.83 (0.71–0.98)*	0.82 (0.69–0.98)*
HD		1.14 (0.99–1.31)	1.01 (0.87–1.18)	0.99 (0.85–1.15)	0.95 (0.81–1.11)	0.94 (0.80–1.10)
High Physical activity[a]	5877					
KDM-BA		0.81 (0.76–0.87)***	0.90 (0.84–0.97)**	0.91 (0.85–0.97)**	0.87 (0.80–0.93)***	0.84 (0.78–0.91)***
Phenoage-BA		1.04 (0.97–1.12)	0.98 (0.91–1.06)	0.99 (0.92–1.07)	0.90 (0.83–0.97)**	0.86 (0.79–0.94)**
HD		1.37 (1.28–1.46)***	1.09 (1.02–1.17)*	1.11 (1.03–1.19)**	1.03 (0.96–1.11)	1.00 (0.93–1.08)
**Transports-related PA**						
None Physical activity	14026	1.00 (Ref)	1.00 (Ref)	1.00 (Ref)	1.00 (Ref)	1.00 (Ref)
Low Physical activity	1965					
KDM-BA		1.02 (0.92–1.13)	1.14 (1.03–1.27)*	1.18 (1.07–1.31)**	1.17 (1.06–1.30)**	1.15 (1.04–1.28)**
Phenoage		1.19 (1.09–1.31)***	1.27 (1.15–1.39)***	1.22 (1.11–1.34)***	1.20 (1.09–1.32)***	1.18 (1.07–1.31)***
HD		1.15 (1.04–1.26)**	1.04 (0.94–1.15)	1.08 (0.98–1.19)	1.06 (0.96–1.17)	1.03 (0.93–1.14)
Middle Physical activity	944					
KDM-BA		1.06 (0.92–1.22)	1.18 (1.03–1.37)*	1.23 (1.07–1.42)**	1.22 (1.05–1.41)**	1.19 (1.03–1.38)*
Phenoage		1.22 (1.05–1.43)*	1.14 (0.97–1.33)	1.19 (1.02–1.40)*	1.14 (0.97–1.34)	1.08 (0.92–1.28)
HD		1.21 (1.06–1.38)**	1.06 (0.92–1.22)	1.10 (0.96–1.27)	1.07 (0.93–1.24)	1.02 (0.89–1.18)
High Physical activity	1427					
KDM-BA		0.99 (0.88–1.11)	1.15 (1.03–1.30)*	1.22 (1.08–1.37)**	1.18 (1.05–1.33)**	1.15 (1.02–1.30)*
Phenoage		1.24 (1.09–1.41)**	1.18 (1.04–1.35)*	1.28 (1.12–1.47)***	1.20 (1.05–1.37)**	1.14 (0.99–1.30)
HD		1.32 (1.18–1.47)***	1.15 (1.02–1.29)*	1.22 (1.09–1.37)***	1.16 (1.03–1.31)*	1.11 (0.98–1.25)
**Recreational-related PA**						
None Physical activity	9746	1.00 (Ref)	1.00 (Ref)	1.00 (Ref)	1.00 (Ref)	1.00 (Ref)
Low Physical activity	2633					
KDM-BA		1.19 (1.09–1.31)***	1.27 (1.15–1.39)***	1.22 (1.11–1.34)***	1.20 (1.09–1.32)***	1.18 (1.07–1.31)***
Phenoage		1.51 (1.37–1.67)***	1.46 (1.32–1.62)***	1.38 (1.24–1.53)***	1.32 (1.19–1.47)***	1.29 (1.16–1.44)***
HD		1.46 (1.34–1.59)***	1.35 (1.23–1.48)***	1.28 (1.16–1.40)***	1.24 (1.13–1.37)***	1.23 (1.11–1.35)***
Middle Physical activity	2008					
KDM-BA		1.20 (1.08–1.33)***	1.30 (1.17–1.44)***	1.23 (1.11–1.38)***	1.21 (1.09–1.35)***	1.19 (1.07–1.33)**
Phenoage		1.63 (1.45–1.83)***	1.54 (1.37–1.73)***	1.41 (1.25–1.58)***	1.34 (1.19–1.52)***	1.32 (1.16–1.49)***
HD		1.54 (1.40–1.70)***	1.35 (1.22–1.49)***	1.24 (1.12–1.38)***	1.21 (1.09–1.34)***	1.18 (1.06–1.31)**
High Physical activity	3975					
KDM-BA		0.97 (0.90–1.05)	1.20 (1.10–1.30)***	1.15 (1.06–1.25)**	1.12 (1.03–1.22)**	1.08 (0.99–1.18)
Phenoage		1.78 (1.63–1.95)***	1.65 (1.50–1.81)***	1.52 (1.38–1.67)***	1.42 (1.29–1.57)***	1.35 (1.22–1.49)***
HD		1.85 (1.72–2.00)***	1.45 (1.31–1.54)***	1.32 (1.21–1.43)***	1.26 (1.15–1.37)***	1.18 (1.09–1.29)***

[a] None Physical activity (0 minutes/week), Low Physical activity (1–149 minutes/week), Middle Physical activity (150–300 minutes/week), and High Physical activity (over 300 minutes/week).

[b] Model 1 is an unadjusted model, Model 2 adjusts age and gender, Model 3 continues to adjust education level, marital status, PIR, and race based on Model 2, Model 4 continues to adjust smoking, drinking, sleep, and sedentary behavior based on Model 3, and Model 5 continues to adjust diabetes, cardiovascular disease, and cancer based on Model 4.

TABLE S2. Odds Ratios and confidence intervals of different types of physical activities in delaying aging[b]

	N = 18362	Adjust Model[b]	P-Value[d]	HD1[c]	HD2[c]	HD3[c]
**Work-related PA**						
None Physical activity[a]	10421	1.00 (Ref)		1.00 (Ref)	1.00 (Ref)	1.00 (Ref)
Low Physical activity[a]	1255					
KDM-BA		1.06 (0.93–1.22)	0.226			
Phenoage		0.94 (0.81–1.08)	0.433			
HD		0.95 (0.84–1.09)	0.928	0.95 (0.82–1.10)	1.02 (0.89–1.16)	-0.025 (-0.076–0.026)
Middle Physical activity[a]	809					
KDM-BA		0.88 (0.75–1.03)	0.214			
Phenoage		0.82 (0.69–0.98)	0.034			
HD		0.94 (0.80–1.10)	0.934	1.17 (0.97–1.42)	0.96 (0.81–1.13)	-0.021 (-0.083–0.409)
High Physical activity[a]	5877					
KDM-BA		0.84 (0.78–0.91)	**0.001**			
Phenoage-BA		0.86 (0.79–0.94)	**0.001**			
HD		1.00 (0.93–1.08)	0.576	1.02 (0.94–1.12)	1.01 (0.93–1.09)	-0.027 (-0.056–0.003)
**Transports-related PA**						
None Physical activity	14026	1.00 (Ref)		1.00 (Ref)	1.00 (Ref)	1.00 (Ref)
Low Physical activity	1965					
KDM-BA		1.15 (1.04–1.28)	0.011			
Phenoage		1.18 (1.07–1.31)	0.018			
HD		1.03 (0.93–1.14)	0.749	1.03 (0.91–1.16)	1.01 (0.90–1.13)	-0.003 (-0.044–0.038)
Middle Physical activity	944					
KDM-BA		1.19 (1.03–1.38)	0.014			
Phenoage		1.08 (0.92–1.28)	0.206			
HD		1.02 (0.89–1.18)	0.512	1.11 (0.93–1.33)	1.07 (0.91–1.25)	-0.002 (-0.082–0.033)
High Physical activity	1427					
KDM-BA		1.15 (1.02–1.30)	0.044			
Phenoage		1.14 (0.99–1.30)	0.091			
HD		1.11 (0.98–1.25)	0.141	1.13 (0.97–1.31)	1.15 (1.00–1.31)	-0.002 (-0.068–0.028)
**Recreational-related PA**						
None Physical activity	9746	1.00 (Ref)		1.00 (Ref)	1.00 (Ref)	1.00 (Ref)
Low Physical activity	2633					
KDM-BA		1.18 (1.07–1.31)	**0.001**			
Phenoage		1.29 (1.16–1.44)	**0.001**			
HD		1.23 (1.11–1.35)	**0.001**	1.34 (1.20–1.50)	1.35 (1.22–1.50)	-0.079 (-0.117– -0.041)
Middle Physical activity	2008					
KDM-BA		1.19 (1.07–1.33)	**0.001**			
Phenoage		1.32 (1.16–1.49)	**0.001**			
HD		1.18 (1.06–1.31)	**0.001**	1.36 (1.19–1.55)	1.36 (1.21–1.52)	-0.069 (-0.112– -0.027)
High Physical activity	3975					
KDM-BA		1.08 (0.99–1.18)	0.081			
Phenoage		1.35 (1.22–1.49)	**0.001**			
HD		1.18 (1.09–1.29)	**0.001**	1.47 (1.32–1.64)	1.36 (1.24–1.50)	-0.096 (-0.130 – -0.062)

[a] None Physical activity (0 minutes/week), Low Physical activity (1–149 minutes/week), Middle Physical activity (150–300 minutes/week), and High Physical activity (over 300 minutes/week).
[b] Adjusted models adjusted for covariates age, sex, education level, marital status, PIR, race, smoking, drinking, sleep, sedentary behavior, diabetes, CVD, and cancer.
[c] HD1 is defined as delayed aging when the HD value is below the 75th percentile, and HD2 is defined as delayed aging when the HD value is below the 65th percentile. HD3 treats HD as a continuous variable and was analyzed using linear regression.
[d] P values that remained significant after Bonferroni correction ($P < 0.0056$) are shown in bold.

TABLE S3. Odds Ratios and confidence intervals of different types of physical activities in delaying aging[c]

	N = 17181	Completed[a]	N = 9095	Male	N = 9267	Female
**Work-related PA**						
None Physical activity[b]	9661	1.00 (Ref)	4519	1.00 (Ref)	5902	1.00 (Ref)
Low Physical activity[b]	1190		646		609	
KDM-BA		1.10 (0.95–1.27)		1.05 (0.87–1.27)		1.07 (0.89–1.30)
Phenoage		0.93 (0.80–1.09)		0.93 (0.76–1.12)		0.95 (0.77–1.18)
HD		0.91 (0.79–1.05)		0.90 (0.75–1.09)		1.02 (0.85–1.22)
Middle Physical activity[b]	763		407		402	
KDM-BA		0.91 (0.77–1.08)		0.87 (0.70–1.10)		0.89 (0.72–1.12)
Phenoage		0.82 (0.68–0.99)*		0.79 (0.62–1.00)*		0.87 (0.68–1.13)
HD		0.97 (0.82–1.16)		0.86 (0.69–1.09)		1.02 (0.82–1.28)
High Physical activity[b]	5567		3523		2354	
KDM-BA		0.85 (0.78–0.92)***		0.83 (0.75–0.92)***		0.85 (0.76–0.95)**
Phenoage-BA		0.84 (0.76–0.91)***		0.90 (0.80–1.01)		0.78 (0.69–0.89)***
HD		1.01 (0.93–1.09)		1.04 (0.94–1.14)		0.93 (0.83–1.03)
**Transports-related PA**						
None Physical activity	13144	1.00 (Ref)	6751	1.00 (Ref)	7275	1.00 (Ref)
Low Physical activity	1830		969		996	
KDM-BA		1.17 (1.05–1.32)**		1.29 (1.11–1.50)**		1.03 (0.89–1.19)
Phenoage		1.16 (1.02–1.32)*		1.15 (0.98–1.36)		1.16 (0.98–1.37)
HD		1.07 (0.95–1.20)		1.01 (0.87–1.18)		1.05 (0.91–1.22)
Middle Physical activity	883		505		439	
KDM-BA		1.17 (1.00–1.37)		1.29 (1.05–1.59)*		1.10 (0.89–1.36)
Phenoage		1.10 (0.92–1.31)		0.95 (0.77–1.18)		1.35 (1.04–1.76)*
HD		1.06 (0.91–1.24)		0.98 (0.80–1.20)		1.09 (0.89–1.35)
High Physical activity	1324		870		557	
KDM-BA		1.14 (1.00–1.31)		1.08 (0.93–1.27)		1.26 (1.04–1.53)*
Phenoage		1.10 (0.95–1.29)		1.10 (0.92–1.31)		1.21 (0.97–1.53)
HD		1.11 (0.97–1.27)		1.01 (0.86–1.18)		1.29 (1.07–1.56)**
**Recreational-related PA**						
None Physical activity	9069	1.00 (Ref)	4557	1.00 (Ref)	5189	1.00 (Ref)
Low Physical activity	2461		1214		1419	
KDM-BA		1.21 (1.09–1.34)***		1.27 (1.10–1.47)**		1.12 (0.98–1.28)
Phenoage		1.24 (1.10–1.39)***		1.37 (1.18–1.60)***		1.23 (1.06–1.43)**
HD		1.20 (1.08–1.34)***		1.21 (1.05–1.39)*		1.24 (1.09–1.42)**
Middle Physical activity	1908		954		1054	
KDM-BA		1.20 (1.06–1.35)**		1.24 (1.06–1.45)**		1.16 (1.00–1.35)
Phenoage		1.34 (1.17–1.53)***		1.35 (1.14–1.60)***		1.30 (1.09–1.56)**
HD		1.19 (1.06–1.33)**		1.22 (1.04–1.43)*		1.15 (0.99–1.33)
High Physical activity	3743		2370		1605	
KDM-BA		1.13 (1.03–1.24)**		1.14 (1.01–1.28)*		1.00 (0.88–1.14)
Phenoage		1.32 (1.19–1.47)***		1.37 (1.21–1.56)***		1.28 (1.10–1.50)**
HD		1.19 (1.08–1.31)***		1.17 (1.04–1.31)**		1.22 (1.07–1.38)**

[a] Completed refers to retaining only samples with missing values for all variables (including covariates, biomarkers, and physical activity data).

[b] None Physical activity (0 minutes/week), Low Physical activity (1–149 minutes/week), Middle Physical activity (150–300 minutes/week), and High Physical activity (over 300 minutes/week).

[c] Adjusted models adjusted for covariates age, sex, education level, marital status, PIR, race, smoking, drinking, sleep, sedentary behavior, diabetes, CVD, and cancer.

TABLE S4. Odds Ratios and confidence intervals of different types of physical activities in delaying aging[b]

	N = 13835	< 65	N = 4527	≥ 65
**Work-related PA**				
None Physical activity	7378	1.00 (Ref)	3043	1.00 (Ref)
Low Physical activity	910		345	
KDM-BA		1.00 (0.87–1.17)		1.23 (0.93–1.65)
Phenoage		0.93 (0.78–1.10)		0.94 (0.73–1.22)
HD		1.00 (0.86–1.16)		0.86 (0.66–1.11)
Middle Physical activity	599		210	
KDM-BA		0.83 (0.69–0.99)*		0.97 (0.70–1.36)
Phenoage		0.79 (0.65–0.97)*		0.92 (0.67–1.28)
HD		0.93 (0.78–1.11)		1.06 (0.77–1.46)
High Physical activity	4948		929	
KDM-BA		0.74 (0.68–0.80)***		1.05 (0.88–1.27)
Phenoage-BA		0.84 (0.76–0.92)***		0.94 (0.79–1.11)
HD		1.02 (0.94–1.11)		1.15 (0.96–1.36)
**Transports-related PA**				
None Physical activity	10281	1.00 (Ref)	3745	1.00 (Ref)
Low Physical activity	1596		369	
KDM-BA		1.07 (0.96–1.20)		1.51 (1.15–2.02)**
Phenoage		1.12 (0.98–1.28)		1.27 (0.99–1.64)
HD		1.02 (0.91–1.14)		1.14 (0.90–1.45)
Middle Physical activity	752		192	
KDM-BA		1.13 (0.96–1.32)		1.26 (0.88–1.85)
Phenoage		1.11 (0.92–1.35)		0.97 (0.70–1.37)
HD		1.08 (0.92–1.27)		0.90 (0.64–1.24)
High Physical activity	1206		221	
KDM-BA		1.06 (0.94–1.21)		1.33 (0.94–1.91)
Phenoage		1.07 (0.92–1.24)		1.53 (1.11–2.15)*
HD		1.12 (0.98–1.27)		1.25 (0.92–1.69)
**Recreational-related PA**				
None Physical activity	6880	1.00 (Ref)	2866	1.00 (Ref)
Low Physical activity	2016		617	
KDM-BA		1.07 (0.96–1.19)		1.51 (1.21–1.90)***
Phenoage		1.19 (1.05–1.35)**		1.59 (1.29–1.96)***
HD		1.22 (1.10–1.36)***		1.37 (1.12–1.67)**
Middle Physical activity	1562		446	
KDM-BA		1.08 (0.96–1.22)		1.30 (1.01–1.69)*
Phenoage		1.28 (1.11–1.48)***		1.41 (1.11–1.79)**
HD		1.21 (1.07–1.36)**		1.27 (1.01–1.59)*
High Physical activity	3377		598	
KDM-BA		0.91 (0.83–1.00)		1.24 (0.99–1.57)
Phenoage		1.24 (1.11–1.38)***		1.75 (1.40–2.18)***
HD		1.25 (1.14–1.37)***		1.43 (1.16–1.75)***

[a] None Physical activity (0 minutes/week), Low Physical activity (1–149 minutes/week), Middle Physical activity (150–300 minutes/week), and High Physical activity (over 300 minutes/week).

[b] Adjusted models adjusted for covariates age, sex, education level, marital status, PIR, race, smoking, drinking, sleep, sedentary behavior, diabetes, CVD, and cancer.

TABLE S5. Odds Ratios and confidence intervals of different types of physical activities in delaying aging[c]

	N = 4932	Chronic[a]	N = 13430	No Chronic[a]
**Work-related PA**				
None Physical activity[b]	3103	1.00 (Ref)	7318	1.00 (Ref)
Low Physical activity[b]	376		879	
KDM-BA		1.02 (0.81–1.29)		1.09 (0.93–1.28)
Phenoage		1.10 (0.87–1.37)		0.87 (0.73–1.04)
HD		0.89 (0.68–1.15)		0.99 (0.90–1.06)
Middle Physical activity[b]	225		584	
KDM-BA		0.83 (0.62–1.11)		0.90 (0.75–1.08)
Phenoage		0.88 (0.66–1.18)		0.79 (0.64–0.98)*
HD		1.13 (0.83–1.54)		0.89 (0.75–1.07)
High Physical activity[b]	1228		4649	
KDM-BA		0.98 (0.84–1.14)		0.82 (0.75–0.90)***
Phenoage-BA		1.03 (0.89–1.19)		0.82 (0.75–0.91)***
HD		1.22 (1.04–1.43)*		0.98 (0.90–1.06)
**Transports-related PA**				
None Physical activity	4065	1.00 (Ref)	9961	1.00 (Ref)
Low Physical activity	415		1550	
KDM-BA		1.14 (0.91–1.42)		1.15 (1.02–1.30)*
Phenoage		1.02 (0.82–1.26)		1.22 (1.06–1.41)**
HD		1.05 (0.82–1.33)		1.03 (0.92–1.15)
Middle Physical activity	183		761	
KDM-BA		1.35 (0.98–1.89)		1.15 (0.98–1.36)
Phenoage		1.02 (0.75–1.40)		1.12 (0.93–1.36)
HD		1.10 (0.77–1.55)		1.01 (0.87–1.19)
High Physical activity	269		1158	
KDM-BA		1.45 (1.10–1.92)**		1.10 (0.96–1.26)
Phenoage		1.27 (0.98–1.66)		1.13 (0.97–1.33)
HD		1.14 (0.85–1.51)		1.13 (0.99–1.29)
**Recreational-related PA**				
None Physical activity	3087	1.00 (Ref)	6659	1.00 (Ref)
Low Physical activity	699		1934	
KDM-BA		1.32 (1.10–1.59)**		1.14 (1.02–1.28)*
Phenoage		1.48 (1.24–1.77)***		1.23 (1.08–1.41)**
HD		1.33 (1.09–1.60)**		1.22 (1.09–1.36)***
Middle Physical activity	485		1523	
KDM-BA		1.34 (1.08–1.67)**		1.15 (1.01–1.30)*
Phenoage		1.37 (1.11–1.68)**		1.33 (1.14–1.55)***
HD		1.24 (0.99–1.55)		1.19 (1.05–1.34)**
High Physical activity	661		3314	
KDM-BA		1.30 (1.07–1.58)**		1.05 (0.96–1.16)
Phenoage		1.57 (1.30–1.89)***		1.31 (1.17–1.47)***
HD		1.29 (1.06–1.58)*		1.20 (1.09–1.32)***

[a] Chronic disease was defined as the presence of any of cardiovascular disease, diabetes, and cancer.
[b] None Physical activity (0 minutes/week), Low Physical activity (1–149 minutes/week), Middle Physical activity (150–300 minutes/week), and High Physical activity (over 300 minutes/week).
[c] Adjusted models adjusted for covariates age, sex, education level, marital status, PIR, race, smoking, drinking, sleep, sedentary behavior, diabetes, CVD, and cancer.

TABLE S6. Odds Ratios and confidence intervals of different types of physical activities in delaying aging (Male)

				KDM-BA	P^b	Phenoage	P^b	HD	P^b
				OR (95%CI)		OR (95%CI)		OR (95%CI)	
OPA[a]-LTPA[a]	Low OPA	Low LTPA	3460	1.00 (Ref)		1.00 (Ref)		1.00 (Ref)	
	High OPA	Low LTPA	2311	0.90 (0.79–1.01)	0.084	0.80 (0.70–0.92)	**0.001**	0.97 (0.86–1.09)	0.594
	High LTPA	Low OPA	1705	1.12 (0.98–1.27)	0.098	1.29 (1.10–1.50)	**0.002**	1.18 (1.04–1.34)	0.009
	High LTPA	High OPA	1619	0.85 (0.73–0.98)	0.029	0.97 (0.81–1.16)	0.714	1.01 (0.87–1.17)	0.927
OPA[a]-TPA[a]	Low OPA	Low TPA	4487	1.00 (Ref)		1.00 (Ref)		1.00 (Ref)	
	High OPA	Low TPA	3233	0.83 (0.74–0.92)	**0.001**	0.78 (0.69–0.88)	**0.001**	0.93 (0.83–1.03)	0.163
	High TPA	Low OPA	679	1.12 (0.92–1.35)	0.256	1.20 (0.96–1.51)	0.111	1.18 (0.98–1.42)	0.079
	High TPA	High OPA	697	1.10 (0.88–1.38)	0.411	1.07 (0.81–1.40)	0.636	1.14 (0.91–1.42)	0.263
TPA[a]-LTPA[a]	Low TPA	Low LTPA	5059	1.00 (Ref)		1.00 (Ref)		1.00 (Ref)	
	High TPA	Low LTPA	712	1.16 (0.97–1.40)	0.108	1.39 (1.11–1.73)	**0.004**	1.25 (1.04–1.49)	0.016
	High LTPA	Low TPA	2661	1.02 (0.91–1.14)	0.725	1.27 (1.11–1.45)	**0.001**	1.14 (1.02–1.27)	0.022
	High LTPA	High TPA	663	1.23 (0.97–1.55)	0.084	1.21 (0.92–1.60)	0.181	1.21 (0.96–1.52)	0.105

[a] Low OPA, Low TPA, LOW LTPA, High OPA, High TPA, and High LTPA were grouped according to whether the weekly physical activity time was less than or equal to 150 minutes.
[b] P values that remained significant after Bonferroni correction ($P < 0.0056$) are shown in bold.

TABLE S7. Odds Ratios and confidence intervals of different types of physical activities in delaying aging (Female)

				KDM-BA	P^b	Phenoage	P^b	HD	P^b
				OR (95%CI)		OR (95%CI)		OR (95%CI)	
OPA[a]-LTPA[a]	Low OPA	Low LTPA	4886	1.00 (Ref)		1.00 (Ref)		1.00 (Ref)	
	High OPA	Low LTPA	1722	0.85 (0.75–0.96)	0.008	0.97 (0.85–1.10)	0.604	1.09 (0.97–1.24)	0.156
	High LTPA	Low OPA	1625	1.16 (1.01–1.33)	0.031	1.46 (1.26–1.69)	**0.001**	1.22 (1.06–1.39)	**0.005**
	High LTPA	High OPA	1034	0.91 (0.79–1.04)	0.169	1.07 (0.92–1.25)	0.366	1.14 (0.99–1.31)	0.073
OPA[a]-TPA[a]	Low OPA	Low TPA	5910	1.00 (Ref)		1.00 (Ref)		1.00 (Ref)	
	High OPA	Low TPA	2361	0.82 (0.74–0.91)	**0.001**	0.90 (0.80–1.01)	0.064	1.07 (0.96–1.19)	0.236
	High TPA	Low OPA	601	1.11 (0.92–1.33)	0.279	1.04 (0.86–1.27)	0.661	1.09 (0.91–1.30)	0.372
	High TPA	High OPA	395	0.93 (0.78–1.12)	0.457	0.91 (0.75–1.10)	0.319	0.97 (0.81–1.16)	0.732
TPA[a]-LTPA[a]	Low TPA	Low LTPA	5981	1.00 (Ref)		1.00 (Ref)		1.00 (Ref)	
	High TPA	Low LTPA	627	1.07 (0.90–1.28)	0.418	1.01 (0.85–1.22)	0.879	1.01 (0.85–1.20)	0.935
	High LTPA	Low TPA	2290	1.09 (0.98–1.21)	0.133	1.28 (1.14–1.45)	**0.001**	1.14 (1.03–1.28)	0.016
	High LTPA	High TPA	369	1.23 (1.02–1.47)	0.030	1.23 (1.00–1.51)	0.049	1.09 (0.90–1.30)	0.379

[a] Low OPA, Low TPA, LOW LTPA, High OPA, High TPA, and High LTPA were grouped according to whether the weekly physical activity time was less than or equal to 150 minutes.
[b] P values that remained significant after Bonferroni correction ($P < 0.0056$) are shown in bold.1

TABLE S8. Odds Ratios and confidence intervals of different types of physical activities in delaying aging (< 65)

				KDM-BA	P[b]	Phenoage	P[b]	HD	P[b]
				OR (95%CI)		OR (95%CI)		OR (95%CI)	
**OPA[a]-LTPA[a]**	Low OPA	Low LTPA	5598	1.00 (Ref)		1.00 (Ref)		1.00 (Ref)	
	High OPA	Low LTPA	3298	0.77 (0.70–0.84)	**0.001**	0.87 (0.78–0.97)	**0.011**	1.04 (0.94–1.14)	0.482
	High LTPA	Low OPA	2690	1.00 (0.90–1.11)	0.966	1.30 (1.15–1.48)	**0.001**	1.23 (1.11–1.36)	**0.001**
	High LTPA	High OPA	2249	0.72 (0.65–0.80)	**0.001**	0.98 (0.86–1.11)	0.734	1.15 (1.02–1.28)	0.017
**OPA[a]-TPA[a]**	Low OPA	Low TPA	7267	1.00 (Ref)		1.00 (Ref)		1.00 (Ref)	
	High OPA	Low TPA	4610	0.73 (0.67–0.79)	**0.001**	0.83 (0.76–0.92)	**0.001**	1.02 (0.94–1.11)	0.574
	High TPA	Low OPA	1021	1.02 (0.88–1.17)	0.826	1.07 (0.90–1.26)	0.457	1.17 (1.01–1.35)	**0.036**
	High TPA	High OPA	937	0.87 (0.75–1.00)	0.057	0.91 (0.77–1.08)	0.291	1.05 (0.91–1.22)	0.504
**TPA[a]-LTPA[a]**	Low TPA	Low LTPA	7829	1.00 (Ref)		1.00 (Ref)		1.00 (Ref)	
	High TPA	Low LTPA	1067	1.03 (0.90–1.19)	0.631	1.06 (0.91–1.25)	0.449	1.10 (0.95–1.26)	0.195
	High LTPA	Low TPA	4048	0.93 (0.85–1.01)	0.082	1.21 (1.09–1.33)	**0.001**	1.17 (1.08–1.28)	**0.001**
	High LTPA	High TPA	891	1.07 (0.92–1.24)	0.381	1.22 (1.02–1.47)	0.028	1.24 (1.07–1.45)	**0.005**

[a] Low OPA, Low TPA, LOW LTPA, High OPA, High TPA, and High LTPA were grouped according to whether the weekly physical activity time was less than or equal to 150 minutes.
[b] P values that remained significant after Bonferroni correction (P < 0.0056) are shown in bold.

TABLE S9. Odds Ratios and confidence intervals of different types of physical activities in delaying aging (≥ 65)

				KDM-BA	P[b]	Phenoage	P[b]	HD	P[b]
				OR (95%CI)		OR (95%CI)		OR (95%CI)	
**OPA[a]-LTPA[a]**	Low OPA	Low LTPA	2748	1.00 (Ref)		1.00 (Ref)		1.00 (Ref)	
	High OPA	Low LTPA	735	1.01 (0.83–1.24)	0.898	0.92 (0.77–1.11)	0.409	1.23 (1.01–1.49)	0.037
	High LTPA	Low OPA	640	1.20 (0.96–1.50)	0.118	1.52 (1.22–1.88)	**0.001**	1.36 (1.12–1.66)	**0.002**
	High LTPA	High OPA	404	1.14 (0.87–1.49)	0.361	1.27 (0.99–1.63)	0.064	1.28 (1.01–1.62)	0.045
**OPA[a]-TPA[a]**	Low OPA	Low TPA	3130	1.00 (Ref)		1.00 (Ref)		1.00 (Ref)	
	High OPA	Low TPA	984	1.03 (0.86–1.23)	0.767	0.92 (0.77–1.08)	0.299	1.14 (0.96–1.35)	0.127
	High TPA	Low OPA	258	1.35 (0.97–1.88)	0.077	1.14 (0.84–1.54)	0.397	1.01 (0.75–1.35)	0.960
	High TPA	High OPA	155	1.11 (0.74–1.66)	0.619	1.24 (0.84–1.82)	0.275	1.24 (0.87–1.77)	0.239
**TPA[a]-LTPA[a]**	Low TPA	Low LTPA	3211	1.00 (Ref)		1.00 (Ref)		1.00 (Ref)	
	High TPA	Low LTPA	272	1.29 (0.94–1.77)	0.110	1.42 (1.06–1.91)	0.019	1.26 (0.95–1.67)	0.106
	High LTPA	Low TPA	903	1.18 (0.97–1.43)	0.092	1.55 (1.29–1.86)	**0.001**	1.35 (1.14–1.61)	**0.001**
	High LTPA	High TPA	141	1.28 (0.82–1.99)	0.275	1.16 (0.78–1.73)	0.470	0.94 (0.64–1.38)	0.741

[a] Low OPA, Low TPA, LOW LTPA, High OPA, High TPA, and High LTPA were grouped according to whether the weekly physical activity time was less than or equal to 150 minutes.
[b] P values that remained significant after Bonferroni correction (P < 0.0056) are shown in bold.

TABLE S10. Odds Ratios and confidence intervals of different types of physical activities in delaying aging (Chronic[b])

			KDM-BA	P[c]	Phenoage	P[c]	HD	P[c]
			OR (95%CI)		OR (95%CI)		OR (95%CI)	
**OPA[a]-LTPA[a]**	Low OPA	Low LTPA	1.00 (Ref)		1.00 (Ref)		1.00 (Ref)	
	High OPA	Low LTPA	0.99 (0.84–1.16)	0.900	1.00 (0.86–1.17)	0.958	1.26 (1.06–1.50)	**0.010**
	High LTPA	Low OPA	1.36 (1.12–1.65)	**0.002**	1.42 (1.18–1.71)	**0.001**	1.23 (1.00–1.50)	0.046
	High LTPA	High OPA	1.07 (0.86–1.34)	0.533	1.25 (1.01–1.56)	0.039	1.35 (1.08–1.70)	0.009
**OPA[a]-TPA[a]**	Low OPA	Low TPA	1.00 (Ref)		1.00 (Ref)		1.00 (Ref)	
	High OPA	Low TPA	0.96 (0.83–1.11)	0.608	1.00 (0.87–1.15)	0.984	1.25 (1.07–1.47)	**0.004**
	High TPA	Low OPA	1.58 (1.18–2.11)	**0.002**	1.23 (0.94–1.61)	0.140	1.23 (0.91–1.67)	0.172
	High TPA	High OPA	1.15 (0.84–1.58)	0.392	1.08 (0.80–1.47)	0.624	1.19 (0.85–1.65)	0.310
**TPA[a]-LTPA[a]**	Low TPA	Low LTPA	1.00 (Ref)		1.00 (Ref)		1.00 (Ref)	
	High TPA	Low LTPA	1.35 (1.04–1.74)	0.023	1.23 (0.96–1.57)	0.103	1.25 (0.96–1.64)	0.100
	High LTPA	Low TPA	1.21 (1.03–1.43)	0.018	1.39 (1.19–1.62)	**0.001**	1.24 (1.05–1.46)	0.013
	High LTPA	High TPA	1.72 (1.17–2.53)	0.006	1.29 (0.91–1.85)	0.156	1.04 (0.70–1.54)	0.857

[a] Low OPA, Low TPA, LOW LTPA, High OPA, High TPA, and High LTPA were grouped according to whether the weekly physical activity time was less than or equal to 150 minutes.
[b] Chronic refers to a person who has Diabetes, Cardiovascular disease(CVD) or Cancer.
[c] P values that remained significant after Bonferroni correction (P < 0.0056) are shown in bold.

TABLE S11. Odds Ratios and confidence intervals of different types of physical activities in delaying aging(No Chronic[b])

			KDM-BA	P[c]	Phenoage	P[c]	HD	P[c]
			OR (95%CI)		OR (95%CI)		OR (95%CI)	
**OPA[a]-LTPA[a]**	Low OPA	Low LTPA	1.00 (Ref)		1.00 (Ref)		1.00 (Ref)	
	High OPA	Low LTPA	0.83 (0.75–0.92)	**0.001**	0.86 (0.77–0.97)	**0.011**	1.00 (0.90–1.10)	0.925
	High LTPA	Low OPA	1.08 (0.97–1.21)	0.145	1.36 (1.19–1.55)	**0.001**	1.20 (1.08–1.33)	**0.001**
	High LTPA	High OPA	0.86 (0.77–0.97)	0.011	1.01 (0.88–1.16)	0.872	1.07 (0.96–1.20)	0.231
**OPA[a]-TPA[a]**	Low OPA	Low TPA	1.00 (Ref)		1.00 (Ref)		1.00 (Ref)	
	High OPA	Low TPA	0.80 (0.73–0.87)	**0.001**	0.82 (0.74–0.90)	**0.001**	0.97 (0.89–1.06)	0.473
	High TPA	Low OPA	1.01 (0.87–1.16)	0.939	1.05 (0.88–1.25)	0.599	1.10 (0.95–1.26)	0.193
	High TPA	High OPA	0.98 (0.84–1.14)	0.773	0.96 (0.80–1.15)	0.656	1.03 (0.88–1.19)	0.749
**TPA[a]-LTPA[a]**	Low TPA	Low LTPA	1.00 (Ref)		1.00 (Ref)		1.00 (Ref)	
	High TPA	Low LTPA	1.05 (0.91–1.21)	0.498	1.14 (0.96–1.34)	0.129	1.10 (0.95–1.26)	0.197
	High LTPA	Low TPA	1.03 (0.94–1.12)	0.532	1.28 (1.15–1.42)	**0.001**	1.15 (1.05–1.25)	**0.002**
	High LTPA	High TPA	1.17 (1.00–1.37)	0.046	1.25 (1.04–1.50)	0.020	1.17 (1.00–1.36)	0.043

[a] Low OPA, Low TPA, LOW LTPA, High OPA, High TPA, and High LTPA were grouped according to whether the weekly physical activity time was less than or equal to 150 minutes.
[b] No Chronic means no diabetes, cardiovascular disease or cancer.
[c] P values that remained significant after Bonferroni correction (P < 0.0056) are shown in bold.

TABLE S12. Odds Ratios and confidence intervals of different types of physical activities in delaying aging (Completed[b])

				KDM-BA	P[c]	Phenoage	P[c]	HD	P[c]
				OR (95%CI)		OR (95%CI)		OR (95%CI)	
OPA[a]-LTPA[a]	Low OPA	Low LTPA	7423	1.00 (Ref)		1.00 (Ref)		1.00 (Ref)	
	High OPA	Low LTPA	3751	0.87 (0.79–0.95)	**0.003**	0.86 (0.78–0.95)	**0.003**	1.05 (0.96–1.16)	0.276
	High LTPA	Low OPA	3084	1.16 (1.05–1.29)	**0.004**	1.35 (1.20–1.52)	**0.001**	1.19 (1.08–1.32)	**0.001**
	High LTPA	High OPA	2496	0.92 (0.82–1.02)	0.113	1.03 (0.91–1.16)	0.663	1.11 (0.99–1.24)	0.063
OPA[a]-TPA[a]	Low OPA	Low TPA	9348	1.00 (Ref)		1.00 (Ref)		1.00 (Ref)	
	High OPA	Low TPA	5233	0.83 (0.76–0.90)	**0.001**	0.84 (0.76–0.91)	**0.001**	1.02 (0.94–1.11)	0.592
	High TPA	Low OPA	1159	1.07 (0.93–1.24)	0.359	1.09 (0.92–1.28)	0.323	1.11 (0.96–1.28)	0.144
	High TPA	High OPA	1014	1.01 (0.87–1.17)	0.919	0.92 (0.78–1.09)	0.343	1.06 (0.91–1.23)	0.447
TPA[a]-LTPA[a]	Low TPA	Low LTPA	9965	1.00 (Ref)		1.00 (Ref)		1.00 (Ref)	
	High TPA	Low LTPA	1209	1.09 (0.95–1.25)	0.233	1.12 (0.96–1.31)	0.139	1.13 (0.98–1.30)	0.086
	High LTPA	Low TPA	4616	1.08 (1.00–1.18)	0.061	1.29 (1.17–1.42)	**0.001**	1.15 (1.06–1.25)	**0.001**
	High LTPA	High TPA	964	1.25 (1.07–1.46)	**0.005**	1.23 (1.03–1.47)	0.022	1.13 (0.97–1.32)	0.120

[a] Low OPA, Low TPA, LOW LTPA, High OPA, High TPA, and High LTPA were grouped according to whether the weekly physical activity time was less than or equal to 150 minutes.
[b] Completed refers to retaining only samples with missing values for all variables (including covariates, biomarkers, and physical activity data).
[c] P values that remained significant after Bonferroni correction (P < 0.0056) are shown in bold.

TABLE S13. Odds Ratios and confidence intervals of different types of physical activities in delaying aging(Model1[a])

			KDM-BA	P[c]	Phenoage	P[c]	HD	P[c]
			OR (95%CI)		OR (95%CI)		OR (95%CI)	
OPA[a]-LTPA[a]	Low OPA	Low LTPA	1.00 (Ref)		1.00 (Ref)		1.00 (Ref)	
	High OPA	Low LTPA	0.96 (0.89–1.04)	0.321	1.02 (0.93–1.11)	0.710	1.18 (1.09–1.28)	**0.001**
	High LTPA	Low OPA	1.28 (1.17–1.40)	**0.001**	1.65 (1.49–1.82)	**0.001**	1.43 (1.31–1.56)	**0.001**
	High LTPA	High OPA	1.03 (0.94–1.14)	0.505	1.32 (1.19–1.47)	**0.001**	1.38 (1.25–1.51)	**0.001**
OPA[a]-TPA[a]	Low OPA	Low TPA	1.00 (Ref)		1.00 (Ref)		1.00 (Ref)	
	High OPA	Low TPA	0.91 (0.84–0.97)	**0.005**	0.98 (0.90–1.05)	0.519	1.14 (1.07–1.23)	**0.001**
	High TPA	Low OPA	1.12 (0.99–1.28)	0.069	1.18 (1.03–1.36)	0.017	1.15 (1.02–1.30)	0.021
	High TPA	High OPA	1.05 (0.92–1.20)	0.470	1.08 (0.93–1.25)	0.338	1.12 (0.98–1.28)	0.085
TPA[a]-LTPA[a]	Low TPA	Low LTPA	1.00 (Ref)		1.00 (Ref)		1.00 (Ref)	
	High TPA	Low LTPA	1.14 (1.00–1.28)	0.041	1.23 (1.08–1.41)	**0.002**	1.15 (1.03–1.30)	0.017
	High LTPA	Low TPA	1.18 (1.10–1.27)	**0.001**	1.54 (1.42–1.67)	**0.001**	1.36 (1.27–1.46)	**0.001**
	High LTPA	High TPA	1.29 (1.12–1.48)	**0.001**	1.42 (1.21–1.66)	**0.001**	1.26 (1.10–1.45)	**0.001**

[a] Low OPA, Low TPA, LOW LTPA, High OPA, High TPA, and High LTPA were grouped according to whether the weekly physical activity time was less than or equal to 150 minutes.
[b] Model 1 adjusted for age and gender.
[c] P values that remained significant after Bonferroni correction (P < 0.0056) are shown in bold.

TABLE S14. Odds Ratios and confidence intervals of different types of physical activities in delaying aging (Model2[a])

			KDM-BA	P[c]	Phenoage	P[c]	HD	P[c]
			OR (95%CI)		OR (95%CI)		OR (95%CI)	
**OPA[a]-LTPA[a]**	Low OPA	Low LTPA	1.00 (Ref)		1.00 (Ref)		1.00 (Ref)	
	High OPA	Low LTPA	0.89 (0.82–0.97)	**0.009**	0.93 (0.85–1.02)	0.104	1.07 (0.98–1.16)	0.121
	High LTPA	Low OPA	1.18 (1.07–1.29)	**0.001**	1.43 (1.28–1.58)	**0.001**	1.24 (1.14–1.36)	**0.001**
	High LTPA	High OPA	0.93 (0.84–1.02)	0.134	1.11 (0.99–1.24)	0.079	1.15 (1.04–1.27)	0.006
**OPA[a]-TPA[a]**	Low OPA	Low TPA	1.00 (Ref)		1.00 (Ref)		1.00 (Ref)	
	High OPA	Low TPA	0.85 (0.79–0.91)	**0.001**	0.89 (0.82–0.97)	**0.005**	1.04 (0.97–1.12)	0.282
	High TPA	Low OPA	1.15 (1.01–1.31)	0.037	1.16 (1.01–1.35)	0.038	1.18 (1.04–1.34)	0.010
	High TPA	High OPA	1.04 (0.90–1.19)	0.619	1.02 (0.88–1.19)	0.779	1.08 (0.94–1.24)	0.258
**TPA[a]-LTPA[a]**	Low TPA	Low LTPA	1.00 (Ref)		1.00 (Ref)		1.00 (Ref)	
	High TPA	Low LTPA	1.14 (1.00–1.29)	0.042	1.19 (1.04–1.37)	0.011	1.15 (1.02–1.30)	0.024
	High LTPA	Low TPA	1.08 (1.00–1.17)	0.038	1.33 (1.22–1.45)	**0.001**	1.19 (1.10–1.28)	**0.001**
	High LTPA	High TPA	1.29 (1.12–1.48)	**0.001**	1.33 (1.13–1.56)	**0.001**	1.22 (1.06–1.40)	0.006

[a] Low OPA, Low TPA, LOW LTPA, High OPA, High TPA, and High LTPA were grouped according to whether the weekly physical activity time was less than or equal to 150 minutes.
[b] Model 2 adjusted for age, sex, PIR, education level, marital status, race, smoking, drinking, sleep, and sedentary behavior.
[c] P values that remained significant after Bonferroni correction (P < 0.0056) are shown in bold.

TABLE S15. Odds Ratios and confidence intervals of different types of physical activities in delaying aging(Physical Activity[a])

				KDM-BA	P[b]	Phenoage	P[b]	HD	P[b]
				OR (95%CI)		OR (95%CI)		OR (95%CI)	
**OPA[a]-LTPA[a]**	No OPA	No LTPA	6088	1.00 (Ref)		1.00 (Ref)		1.00 (Ref)	
	OPA	No LTPA	3658	0.93 (0.85–1.02)	0.151	0.88 (0.79–0.97)	0.008	1.02 (0.93–1.12)	0.617
	LTPA	No OPA	4333	1.23 (1.12–1.35)	**0.001**	1.38 (1.25–1.53)	**0.001**	1.26 (1.15–1.38)	**0.001**
	LTPA	OPA	4283	1.00 (0.91–1.09)	0.931	1.13 (1.02–1.25)	0.021	1.16 (1.05–1.27)	**0.002**
**OPA[a]-TPA[a]**	No OPA	No TPA	8161	1.00 (Ref)		1.00 (Ref)		1.00 (Ref)	
	OPA	No TPA	5865	0.88 (0.81–0.95)	**0.001**	0.84 (0.77–0.91)	**0.001**	0.97 (0.90–1.05)	0.496
	TPA	No OPA	2260	1.16 (1.04–1.29)	0.006	1.06 (0.95–1.20)	0.303	1.03 (0.93–1.15)	0.543
	TPA	OPA	2076	1.02 (0.92–1.14)	0.689	1.02 (0.90–1.16)	0.714	1.05 (0.94–1.17)	0.397
**TPA[a]-LTPA[a]**	No TPA	No LTPA	7733	1.00 (Ref)		1.00 (Ref)		1.00 (Ref)	
	TPA	No LTPA	2013	1.17 (1.05–1.31)	**0.004**	1.23 (1.09–1.38)	**0.001**	1.12 (1.00–1.25)	0.041
	LTPA	No TPA	6293	1.14 (1.06–1.23)	**0.001**	1.38 (1.27–1.51)	**0.001**	1.24 (1.15–1.33)	**0.001**
	LTPA	TPA	2323	1.28 (1.15–1.42)	**0.001**	1.36 (1.20–1.53)	**0.001**	1.19 (1.08–1.33)	**0.001**

[a] According to whether they participated in three types of physical activities every week, they were divided into No OPA, No TPA, No LTPA, OPA, TPA, and LTPA.
[b] P values that remained significant after Bonferroni correction (P < 0.0056) are shown in bold.

TABLE S16. Interaction analysis of different PA with Biological age among participants

	Additive interactive[a]				Multiplicative interactive[a] OR (95%CI)	P value[a]
	Measure	Estimate	Lower	Upper		
**OPA-LTPA**	RERI	-0.129	-0.213	-0.044		
KDM-BA	AP	-0.147	-0.264	-0.029	0.897 (0.781–1.030)	0.125
	S	-16.485	NaN	NaN		
	RERI	0.006	-0.151	0.163		
Phenoage	AP	0.005	-0.118	0.128	0.849 (0.725–0.995)	**0.043**
	S	1.023	0.571	1.832		
	RERI	0.103	-0.047	0.254		
HD	AP	0.078	-0.022	0.177	0.873 (0.760–1.003)	0.055
	S	1.454	0.997	2.121		
**OPA-TPA**	RERI	-0.020	-0.148	0.109		
KDM-BA	AP	-0.022	-0.165	0.122	1.088 (0.899–1.317)	0.387
	S	1.312	0.255	6.752		
	RERI	-0.049	-0.190	0.091		
Phenoage	AP	-0.055	-0.220	0.111	1.030 (0.831–1.276)	0.790
	S	1.924	0.221	16.715		
	RERI	0.028	-0.136	0.192		
HD	AP	0.024	-0.114	0.162	0.908 (0.751–1.098)	0.319
	S	1.221	0.439	3.397		
**TPA-LTPA**	RERI	0.277	0.053	0.500		
KDM-BA	AP	0.192	0.075	0.310	1.055 (0.870–1.280)	0.587
	S	2.710	1.408	5.217		
	RERI	0.383	0.051	0.715		
Phenoage	AP	0.212	0.077	0.347	0.844 (0.678–1.052)	0.132
	S	1.906	1.326	2.738		
	RERI	0.173	-0.042	0.388		
HD	AP	0.122	-0.006	0.250	0.894 (0.738–1.083)	0.253
	S	1.708	1.055	2.766		

[a] All additive and multiplicative interaction analyses above were adjusted for age, sex, education level, marital status, PIR, race, smoking, alcohol consumption, sleep, sedentary behavior, diabetes, cardiovascular disease, and cancer.

TABLE S17. Odds Ratios and confidence intervals of Occupational physical activity in delaying aging[b]

	N = 18362	SES3[b]	P-Value[c]	SES4[b]	P-Value[c]
**Work-related PA**					
None Physical activity[a]	10421	1.00 (Ref)		1.00 (Ref)	
Low Physical activity[a]	1255				
KDM-BA		1.09 (0.95–1.24)	0.218	1.09 (0.96–1.25)	0.177
Phenoage		0.93 (0.81–1.07)	0.331	0.95 (0.82–1.09)	0.454
HD		1.01 (0.89–1.14)	0.901	1.02 (0.90–1.16)	0.788
Middle Physical activity[a]	809				
KDM-BA		0.91 (0.78–1.07)	0.258	0.92 (0.79–1.08)	0.301
Phenoage		0.84 (0.71–1.00)	0.049	0.85 (0.72–1.00)	0.055
HD		1.01 (0.87–1.18)	0.858	1.02 (0.87–1.19)	0.828
High Physical activity[a]	5877				
KDM-BA		0.84 (0.78–0.90)	**0.001**	0.84 (0.78–0.90)	0.001
Phenoage-BA		0.82 (0.76–0.89)	**0.001**	0.82 (0.76–0.90)	**0.001**
HD		1.01 (0.94–1.08)	0.830	1.01 (0.94–1.08)	0.869

[a] None Physical activity (0 minutes/week), Low Physical activity (1–149 minutes/week), Middle Physical activity (150–300 minutes/week), and High Physical activity (over 300 minutes/week).
[b] Adjusted models adjusted for covariates age, sex, marital status, SES3 or SES4, race, smoking, drinking, sleep, sedentary behavior, diabetes, CVD, and cancer.
[c] P values that remained significant after Bonferroni correction (P < 0.0056) are shown in bold.

TABLE S18. Odds Ratios and confidence intervals of Occupational physical activity in delaying aging[b]

	N = 9816	Low[b]	P-Value[c]
**Work-related PA**			
None Physical activity[a]	5394	1.00 (Ref)	
Low Physical activity[a]	763		
KDM-BA		1.14 (0.96–1.36)	0.138
Phenoage		1.06 (0.87–1.28)	0.579
HD		1.04 (0.88–1.22)	0.653
Middle Physical activity[a]	476		
KDM-BA		0.83 (0.75–1.14)	0.471
Phenoage		0.81 (0.64–1.01)	0.065
HD		1.05 (0.85–1.29)	0.663
High Physical activity[a]	3183		
KDM-BA		0.79 (0.71–0.87)	**0.001**
Phenoage-BA		0.83 (0.74–0.93)	**0.001**
HD		0.99 (0.89–1.10)	0.843
	**N = 4633**	**Medium[b]**	**P-Value[c]**
**Work-related PA**			
None Physical activity[a]	2302	1.00 (Ref)	
Low Physical activity[a]	237		
KDM-BA		0.86 (0.65–1.14)	0.297
Phenoage		0.84 (0.61–1.15)	0.266
HD		0.87 (0.66–1.15)	0.332
Middle Physical activity[a]	148		
KDM-BA		0.90 (0.64–1.28)	0.566
Phenoage		0.95 (0.64–1.42)	0.801
HD		0.93 (0.66–1.31)	0.669
High Physical activity[a]	1946		
KDM-BA		0.91 (0.79–1.03)	0.146
Phenoage-BA		0.87 (0.75–1.02)	0.080
HD		1.05 (0.92–1.19)	0.511
	**N = 3913**	**High[b]**	**P-Value[c]**
**Work-related PA**			
None Physical activity[a]	2725	1.00 (Ref)	
Low Physical activity[a]	255		
KDM-BA		1.20 (0.89–1.60)	0.229
Phenoage		0.81 (0.62–1.07)	0.144
HD		1.07 (0.80–1.42)	0.652
Middle Physical activity[a]	185		
KDM-BA		0.89 (0.64–1.23)	0.473
Phenoage		0.90 (0.65–1.25)	0.528
HD		1.01 (0.73–1.41)	0.930
High Physical activity[a]	748		
KDM-BA		0.86 (0.72–1.03)	0.098
Phenoage-BA		0.74 (0.62–0.88)	**0.001**
HD		0.96 (0.80–1.15)	0.656

[a] None Physical activity (0 minutes/week), Low Physical activity (1–149 minutes/week), Middle Physical activity (150–300 minutes/week), and High Physical activity (over 300 minutes/week).

[b] nalyses were stratified by SES3 and adjusted for age, sex, marital status, race, smoking, alcohol consumption, sleep, sedentary behavior, diabetes, cardiovascular disease, and cancer.

[c] P values that remained significant after Bonferroni correction ($P < 0.0056$) are shown in bold.

TABLE S19. Odds Ratios and confidence intervals of Occupational physical activity in delaying aging[b]

	N = 4070	Low[b]	P-Value[c]	N = 3761	Medium-Low[b]	P-Value[c]
**Work-related PA**						
None Physical activity[a]	1973	1.00 (Ref)		2354	1.00 (Ref)	
Low Physical activity[a]	200			290		
KDM-BA		1.00 (0.73–1.37)	0.988		1.07 (0.81–1.42)	0.620
Phenoage		0.73 (0.51–1.03)	0.070		1.35 (1.01–1.79)	0.041
HD		0.96 (0.71–1.30)	0.792		0.93 (0.71–1.22)	0.580
Middle Physical activity[a]	122			174		
KDM-BA		0.86 (0.59–1.27)	0.459		1.11 (0.78–1.59)	0.548
Phenoage		0.83 (0.53–1.29)	0.402		1.16 (0.81–1.66)	0.424
HD		0.94 (0.64–1.39)	0.761		1.28 (0.91–1.80)	0.151
High Physical activity[a]	1775			943		
KDM-BA		0.94 (0.82–1.09)	0.430		0.99 (0.83–1.18)	0.890
Phenoage-BA		0.84 (0.71–0.99)	0.040		0.92 (0.77–1.10)	**0.359**
HD		1.06 (0.92–1.23)	0.408		1.06 (0.89–1.26)	0.541
	**N = 7226**	**Medium-High[b]**	**P-Value[c]**	**N = 3305**	**High[b]**	**P-Value[c]**
**Work-related PA**						
None Physical activity[a]	3941	1.00 (Ref)		2153	1.00 (Ref)	
Low Physical activity[a]	552			213		
KDM-BA		1.06 (0.87–1.30)	0.572		1.29 (0.95–1.75)	0.104
Phenoage		0.88 (0.70–1.10)	0.262		0.86 (0.64–1.17)	0.336
HD		1.11 (0.91–1.35)	0.302		0.94 (0.69–1.29)	0.708
Middle Physical activity[a]	358			155		
KDM-BA		0.85 (0.67–1.08)	0.179		0.91 (0.64–1.28)	0.572
Phenoage		0.62 (0.47–0.80)	0.001		1.13 (0.78–1.63)	0.518
HD		0.93 (0.74–1.18)	0.559		0.97 (0.68–1.38)	0.866
High Physical activity[a]	2375			784		
KDM-BA		0.73 (0.65–0.82)	**0.001**		0.79 (0.67–0.95)	0.010
Phenoage-BA		0.76 (0.66–0.88)	**0.001**		0.80 (0.66–0.96)	0.017
HD		0.95 (0.85–1.07)	0.425		0.96 (0.80–1.15)	0.664

[a] None Physical activity (0 minutes/week), Low Physical activity (1–149 minutes/week), Middle Physical activity (150–300 minutes/week), and High Physical activity (over 300 minutes/week).
[b] nalyses were stratified by SES4 and adjusted for age, sex, marital status, race, smoking, alcohol consumption, sleep, sedentary behavior, diabetes, cardiovascular disease, and cancer.
[c] P values that remained significant after Bonferroni correction (P < 0.0056) are shown in bold.

Articles published in the Biology of Sport are licensed under an open access Creative Commons CC BY 4.0 license.
